# High-affinity CD16A polymorphism associated with reduced risk ofsevere COVID-19

**DOI:** 10.1172/jci.insight.191314

**Published:** 2025-05-22

**Authors:** Anita E. Qualls, Tasha Tsao, Irene Lui, Shion A. Lim, Yapeng Su, Ernie Chen, Dylan Duchen, Holden T. Maecker, Seunghee Kim-Schulze, Ruth R. Montgomery, Florian Krammer, Charles R. Langelier, Ofer Levy, Lindsey R. Baden, Esther Melamed, Lauren I.R. Ehrlich, Grace A. McComsey, Rafick P. Sekaly, Charles B. Cairns, Elias K. Haddad, Albert C. Shaw, David A. Hafler, David B. Corry, Farrah Kheradmand, Mark A. Atkinson, Scott C. Brakenridge, Nelson I. Agudelo Higuita, Jordan P. Metcalf, Catherine L. Hough, William B. Messer, Bali Pulendran, Kari C. Nadeau, Mark M. Davis, Ana Fernandez-Sesma, Viviana Simon, Monica Kraft, Christian Bime, Carolyn S. Calfee, David J. Erle, Joanna Schaenmann, Al Ozonoff, Bjoern Peters, Steven H. Kleinstein, Alison D. Augustine, Joann Diray-Arce, Patrice M. Becker, Nadine Rouphael, IMPACC Network, Jason D. Goldman, Daniel R. Calabrese, James R. Heath, James A. Wells, Elaine F. Reed, Lewis L. Lanier, Harry Pickering, Oscar A. Aguilar

**Affiliations:** 1 Department of Microbiology and Immunology, UCSF, San Francisco, California, USA.; 2 Department of Pharmaceutical Chemistry, Department of Cellular and Molecular Pharmacology, and Chan Zuckerberg Biohub, UCSF, San Francisco, California, USA.; 3 Institute for Systems Biology, Seattle, Washington, USA.; 4 Yale School of Medicine, New Haven, Connecticut, USA.; 5 Stanford University School of Medicine, Palo Alto, California, USA.; 6 Icahn School of Medicine at Mount Sinai, New York, New York, USA.; 7 Department of Medicine, UCSF, San Francisco, California, USA.; 8 Precision Vaccines Program, Boston Children’s Hospital, and; 9 Brigham and Women’s Hospital, Harvard Medical School, Boston, Massachusetts, USA.; 10 The University of Texas at Austin, Austin, Texas, USA.; 11 Case Western Reserve University and University Hospitals of Cleveland, Cleveland, Ohio, USA.; 12 Drexel University, Tower Health Hospital, Philadelphia, Pennsylvania, USA.; 13 Baylor College of Medicine and the Michael E. DeBakey VA Center for Translational Research on Inflammatory Diseases, Houston, Texas, USA.; 14 University of Florida, Gainesville, Florida, USA.; 15 Oklahoma University Health Sciences Center, Oklahoma City, Oklahoma, USA.; 16 Oregon Health & Science University, Portland, Oregon, USA.; 17 University of Arizona, Tucson, Arizona, USA.; 18 David Geffen School of Medicine at the University of California Los Angeles, Los Angeles, California, USA.; 19 Clinical and Data Coordinating Center (CDCC), Boston Children’s Hospital, Boston, Massachusetts, USA.; 20 La Jolla Institute for Immunology, La Jolla, California, USA.; 21 National Institute of Allergy and Infectious Diseases, NIH, Bethesda, Maryland, USA.; 22 Emory School of Medicine, Atlanta, Georgia, USA.; 23 The Immunophenotyping Assessment in a COVID-19 cohort (IMPACC) Network is detailed in Supplemental Acknowledgments.; 24 Swedish Center for Research and Innovation, Providence Swedish Medical Center, Seattle, Washington, USA.; 25 Division of Allergy and Infectious Diseases, University of Washington, Seattle, Washington, USA.; 26 Parker Institute for Cancer Immunotherapy, San Francisco, California, USA.

**Keywords:** COVID-19, Immunology, Cellular immune response, Innate immunity, NK cells

## Abstract

CD16A is an activating Fc receptor on NK cells that mediates antibody-dependent cellular cytotoxicity (ADCC), a key mechanism in antiviral immunity. However, the role of NK cell–mediated ADCC in SARS-CoV-2 infection remains unclear, particularly whether it limits viral spread and disease severity or contributes to the immunopathogenesis of COVID-19. We hypothesized that the high-affinity CD16A^V176^ polymorphism influences these outcomes. Using an in vitro reporter system, we demonstrated that CD16A^V176^ is a more potent and sensitive activator than the common CD16A^F176^ allele. To assess its clinical relevance, we analyzed 1,027 patients hospitalized with COVID-19 from the Immunophenotyping Assessment in a COVID-19 cohort (IMPACC), a comprehensive longitudinal dataset with extensive transcriptomic, proteomic, and clinical data. The high-affinity CD16A^V176^ allele was associated with a significantly reduced risk of ICU admission, mechanical ventilation, and severe disease trajectories. Lower anti–SARS-CoV-2 IgG titers were correlated to CD16A^V176^; however, there was no difference in viral load across CD16A genotypes. Proteomic analysis revealed that participants homozygous for CD16A^V176^ had lower levels of inflammatory mediators. These findings suggest that CD16A^V176^ enhances early NK cell–mediated immune responses, limiting severe respiratory complications in COVID-19. This study identifies a protective genetic factor against severe COVID-19, informing future host-directed therapeutic strategies.

## Abstract 2

We found that a high affinity version of CD16, a receptor on natural killer cells, confers protection from severe COVID-19 and the associated respiratory sequelae.

## Graphical abstract

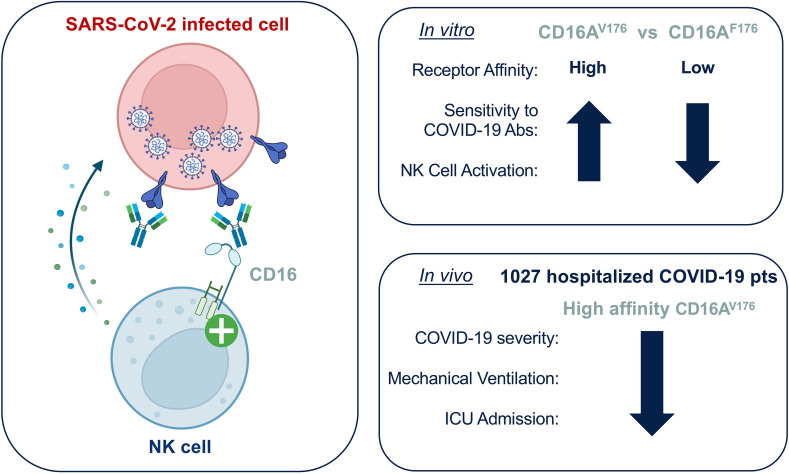

## Introduction

NK cells are lymphocytes of the innate immune system recognized for their early detection and control of viral infection. Using a myriad of receptors, NK cells recognize and bind to ligands or opsonized antibodies on virally infected cells, resulting in their destruction (1). The clearance of antibody-coated cells, also called antibody-dependent cellular cytotoxicity (ADCC), is an important NK cell antiviral mechanism mediated by the activating Fc receptor CD16A (FcγRIIIA, *FCGR3A*) (2–5). Upon CD16A recognition of the IgG-Fc portion, NK cells become activated and release cytolytic mediators including perforin and cytokines such as IFN-γ, resulting in target cell apoptosis. CD16A activation is dependent on immunoreceptor tyrosine-based activation motif–mediated (ITAM-mediated) signaling through CD3ζ or FcεR1γ adaptor molecules (1, 6–9). While ADCC inherently functions to protect the host from infectious pathogens, there has been much debate about whether this host mechanism functions solely in a protective manner or whether it at times may contribute to the proinflammatory pathogenesis of a variety of infectious diseases (10–15).

In the context of SARS-CoV-2 infection, the precise role of NK cell activity remains unclear. Decreased NK cell frequencies in the blood of patients with COVID-19 have been consistently reported (16–25), suggesting NK cell trafficking to the site of infection to mediate antiviral defense (26, 27). In addition, several groups have reported increases in adaptive NK cells (NKG2C^+^CD57^+^FcεR1γ^–^) (16, 19), which are known to have enhanced ADCC capacity (28); however, other groups found no change in this population’s frequency (18, 29). NK cells from patients with severe COVID-19 are extensively altered as they phenotypically and transcriptionally appear highly activated (increased HLA-DR, CD38, CD69 expression) and dysfunctional (increased PD-1 and TIM-3 expression), yet they are more armed (increased perforin and granzyme B) (16, 18, 20, 21). Paradoxically, in vitro studies show NK cells from patients with severe COVID-19 have attenuated cytotoxic potential, including decreased degranulation (measured by CD107a) and less IFN-γ and TNF-α release (19, 21, 23). Several known factors contribute to this NK cell dysfunction, including downregulation of activating receptors NKG2D and DNAM-1 (18, 20, 30) and upregulation of inhibitory NKG2A (22, 29), as well as effects of the cytokine milieu characteristic of severe COVID-19, including NK cell inhibition by TGF-β (23), and chronic stimulation to a state of exhaustion by IFN-α (21). In contrast, several groups have found that early strong NK cell effector function can robustly limit SARS-CoV-2 viral burden and disease (31, 32). Overall, NK cell–mediated cytotoxicity is negatively correlated with COVID-19 severity (21, 23, 29). While it is known that patients with severe SARS-CoV-2 have NK cells that are poor mediators of cytotoxicity, it remains unclear if this is due solely to the cytokine and viral modulation factors already described or if their NK cells from these individuals indeed have basal deficiencies in effector function that initially limited viral control.

In regard to NK cell–mediated ADCC in COVID-19, it is now established that SARS-CoV-2 antibodies generated from infection and/or vaccination can mediate ADCC and improve viral clearance in vitro (33–37). Several groups conducted assays that found elevated ADCC with serum from patients with severe COVID-19 compared with patients with mild infections (32, 38, 39). However, upon analysis of patients with severe COVID-19 separately, higher ADCC activity was displayed in assays with serum from those who survived compared with those who died (32). Interestingly, Adeniji et al. (40) reported that ADCC results varied by antigen, as they noted higher S1-specific ADCC in mild versus severe patients, while the opposite was true for receptor binding domain–specific (RBD-specific) ADCC. Overall, the nuances of Fc effector functions in COVID-19 remain to be fully elucidated.

There is a lack of consensus on whether ADCC primarily limits SARS-CoV-2 spread and disease severity or contributes to the immunopathogenesis of severe COVID-19. A critical gap in the research on ADCC in COVID-19 is the contribution of host genetic variants in NK cell receptors. Importantly, there is a single-nucleotide polymorphism in the *FCGR3A* gene (NM_000569.8:c526T>G), resulting in low affinity phenylalanine (F) or high-affinity valine (V) at amino acid position 176 (sometimes referred to as polymorphism at position 158). CD16A^V176^ is a minor allele that has previously been shown to have greater affinity for all human IgG subclasses (41, 42).

The role of ADCC in viral control is well established in HIV, where elite controllers—individuals who suppress HIV replication without antiretroviral therapy—exhibit strong NK cell–mediated ADCC (43). Studies have shown that the high-affinity CD16A^V176^ allele is enriched in these individuals and correlates with more effective NK cell responses (44). This CD16A polymorphism has clinical relevance in antibody-based therapies as well. The CD16A^V176^ allele is associated with better clinical responses to rituximab in follicular lymphoma due to enhanced NK cell–mediated ADCC (45, 46). Patients carrying the CD16A^V176^ allele demonstrated stronger rituximab binding, increased NK cell activation, and improved progression-free survival compared with those homozygous for the lower-affinity CD16A^F176^ variant. To our knowledge, only 1 group has investigated this CD16A allotypic variant in a small cohort of Austrian patients with COVID-19 (36) and found that CD16A^V176^ was overrepresented in hospitalized and deceased patients.

In this study, we aimed to better define NK cell–mediated ADCC in the context of SARS-CoV-2 and assess the contributions of this CD16A polymorphism to COVID-19 disease control and/or pathogenesis. To determine associations between the CD16A polymorphism and COVID-19 disease course, we performed a retrospective analysis of 1,027 patients from a comprehensive longitudinal study designated the Immunophenotyping Assessment in a COVID-19 cohort (IMPACC) (47). This study collected and analyzed clinical and laboratory data from unvaccinated patients hospitalized with COVID-19 across the United States to identify immunologic, virologic, proteomic, metabolomic, and genomic features of COVID-19–related severity, susceptibility, and disease course trajectories. IMPACC has already performed deep immunophenotyping on 540 hospitalized patients (48) and identified biological states associated with disease severity, which we referred to and used here in our investigation. Overall, there are very little data on CD16A polymorphisms in COVID-19 and the nuances of its effect on disease course are not fully understood. We developed a robust in vitro system to model ADCC function and evaluated a large longitudinal clinical dataset of patients with COVID-19 to tease apart protective versus pathogenic responses through CD16A allelic differences.

## Results

### Generation of human Fcγ reporter cell lines.

It has been well documented that infection with SARS-CoV-2 drives the generation of virus-specific antibodies (49–51). To determine if these antibodies were capable of triggering Fcγ receptors, we generated chimeric receptors expressing the extracellular domain of the CD16A common low-affinity allele or high-affinity alleles (CD16A^F176^ or CD16A^V176^, respectively) and fused them to a construct expressing a mouse CD8 transmembrane domain and the intracellular domain of mouse CD3ζ. Vectors expressing these constructs were used to transduce BWZ.36 (BWZ) cells to generate reporter cell lines of these Fcγ receptors. Briefly, BWZ cells are a mouse T cell lymphoma that contains a *LacZ* cassette under an NFAT promoter thereby producing β-galactosidase upon activation of ITAM-bearing chimeric receptors (52). These BWZ.CD16A reporters expressed high levels of cell-surface CD16A (Supplemental Figure 1A; supplemental material available online with this article; https://doi.org/10.1172/jci.insight.191314DS1). We tested the ability of these reporters to detect IgGs by absorbing human, mouse, and rat IgGs onto absorbent plates, and we then cultured the BWZ reporters in these wells (Supplemental Figure 1B). The CD16A reporters were capable of detecting plate-bound IgGs from expected IgG classes (53). Consistent with previous studies, CD16A detected all human IgGs, but in our system, IgG3 isotypes produced weaker signals and IgG2 antibodies delivered stronger signals than measurements using surface plasmon resonance technique to detect binding of these isotypes (53). In addition, the lower-affinity CD16A^F176^ reporters could detect mouse IgG2a and rat IgG2b isotypes, while the higher-affinity CD16A^V176^ reporters also detected mouse IgG3 (Supplemental Figure 1B). These results confirmed that our BWZ reporter cells expressing chimeric Fcγ receptors accurately detected the Fc portion of IgG isotype antibodies.

### Development of CELLISA assay to detect CD16A binding to anti–SARS-CoV-2 antibodies.

**Figure 1:**
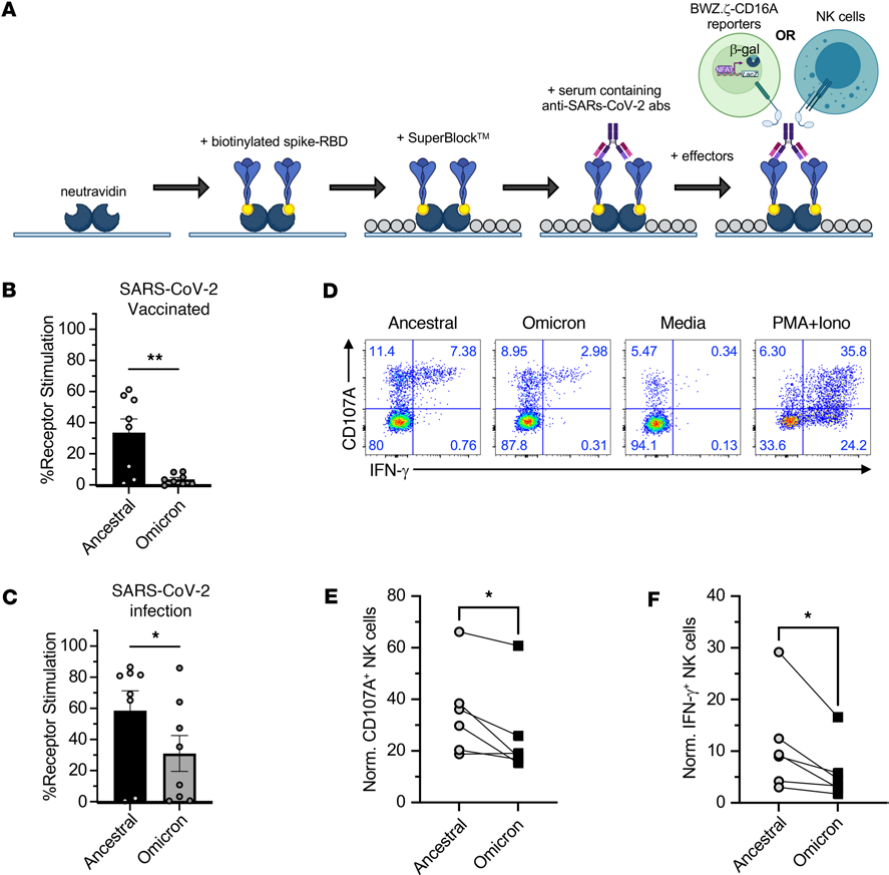
Development of CELLISA assay to measure CD16A responses to antibodies from plasma. (**A**) Schematic of CELLISA assay showing the steps involved to perform functional assays to measure anti-Spike RBD antibodies using BWZ.CD16A reporters or NK cells. (**B** and **C**) Functional assays using BWZ.CD16A^F176^ stimulated using plasma from vaccinated healthy donors (**B**) or acute patients with COVID-19 (**C**) incubated with Ancestral or Omicron Spike RBD. (**D**–**F**) Stimulation of NK cells from PBMCs cultured overnight in IL-2 with plasma from vaccinated donor plasma incubated with Ancestral or Omicron Spike RBD. Representative flow plots (**D**) and quantification of degranulation as measured by CD107A^+^ (**E**) and IFN-γ production (**F**). Data in **E** and **F** are mean ± SEM, each dot representative of replicates (*n* = 6–8). Data were analyzed using a 2-tailed Student’s test (*P < 0.05).

We developed an ELISA-based reporter cell assay as previously described to assess antibody-mediated stimulation (54, 55). Briefly, we first coated high-binding chemistry plates with NeutrAvidin followed by purified biotinylated RBD of SARS-CoV-2 spike protein, blocked with blocking reagent, and incubated with anti–SARS-CoV-2 RBD monoclonal antibodies or patient plasma containing anti–SARS-CoV-2 antibodies. Then, BWZ.CD16A cell reporters were used to assess antibody-mediated stimulation (Figure 1A). As a positive control, we used a monoclonal human IgG1 anti–SARS-CoV-2 Spike RBD antibody that was identified by a phage display screen (Supplemental Figure 1C) (55). Our results demonstrate the sensitivity of our CELLISA technique to distinguish differences between the F176 and V176 allelic variants.

### CD16A reporter stimulation correlates with NK cell function.

The first objective was to confirm that the read-outs of our reporter assays correlated with functional responses mediated by NK cells. Here, we stimulated BWZ.CD16A^F176^ using plasma from healthy donors collected 2–3 months after second SARS-CoV-2 mRNA vaccination (Pfizer-BioNTech BNT162b2 or Moderna mRNA-1273) and tested the ability of our reporters to detect antibody bound to Ancestral or Omicron spike RBD (B.1.1.529) (Figure 1B). Consistent with previous reports, antibodies generated by vaccination against ancestral SARS-CoV-2 are poor at cross-reacting with Omicron RBD. Similar results were also observed with plasma from acutely infected patients (Figure 1C), although these antibodies had more cross-reactivity against Omicron RBD. We proceeded to confirm if this correlated with functional responses in NK cells. Here, plates were coated with spike RBD (Ancestral or Omicron), plasma from a SARS-CoV-2 Ancestral RBD-vaccinated donor were added, and then peripheral blood NK cells, preincubated in recombinant human IL-2 overnight, were placed in the wells. We found that, similar to results with our BWZ.CD16A reporters, NK cells were more strongly activated (as measured by CD107A and IFN-γ production) in wells containing Ancestral RBD in comparison with those containing Omicron RBD (Figure 1, D–F). These results confirm that our reporter readouts correlate with CD16A-mediated responses observed in NK cells.

### The high-affinity CD16A allele is more sensitive at recognizing antibodies generated through SARS-CoV-2 vaccination or infection.

**Figure 2:**
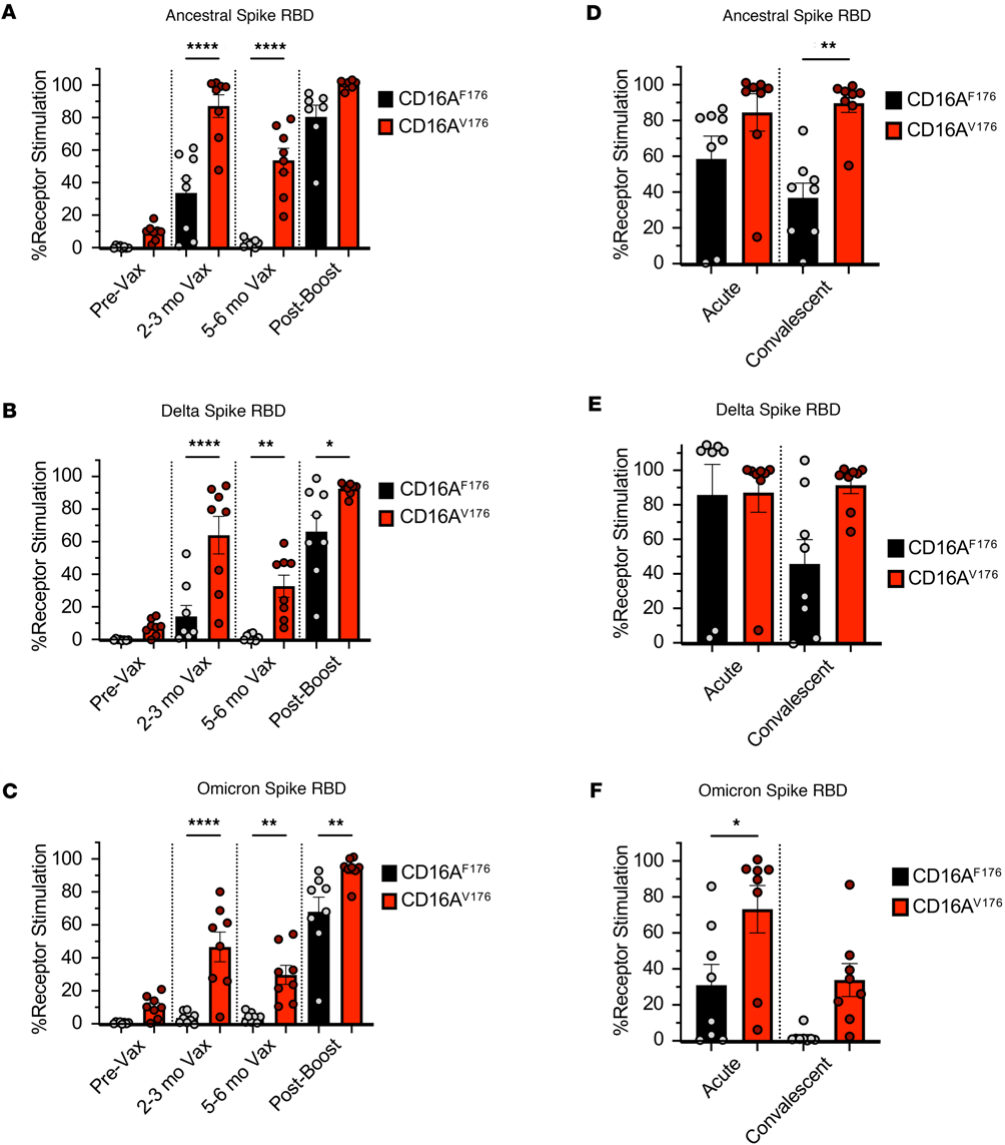
The high affinity CD16A allele is more sensitive at recognizing antibodies generated through SARS-CoV-2 vaccination or infection. (**A**–**C**) Functional assays using BWZ.CD16A^F176^ and BWZ.CD16A^V176^ stimulated by plasma from vaccinated healthy donors at different time points. Data shown are results using Ancestral spike RBD (**A**), Delta spike RBD (**B**), and Omicron spike RBD (**C**). (**D**–**F**) Functional assays using BWZ.CD16A^F176^ and BWZ.CD16A^V176^ stimulated by plasma from patients with COVID-19 at acute (10–14 days following clinical diagnosis) and convalescent (2–3 months after initial onset of symptoms) time points. Data shown are results using ancestral spike RBD (**D**), Delta spike RBD (**E**), and Omicron spike RBD (**F**). Data are shown as mean ± SEM with each dot representing biological replicates (*n* = 8). Data were analyzed using 2-way ANOVA. *P < 0.0332; **P < 0.0021; ****P < 0.0001.

Following the robust validation of our assay, we proceeded to closely monitor the kinetic responses of CD16A reporters to antibodies generated during SARS-CoV-2 vaccination. We hypothesized that allelic differences would be appreciable. Plasma from healthy donors were collected at distinct time points before and after vaccination and was used to assess the ability of BWZ.CD16A reporters to detect plate-bound Spike RBD protein. We observed that sufficient anti-Spike RBD antibodies could be detected in our assay 2–3 months following the second COVID-19 vaccination. However, CD16A activation of the reporters was no longer detectable by 5–6 months after the second vaccination. One to 4 weeks following the subsequent booster shot, there was abundant anti-RBD antibody in the plasma from donors as detected by the strong CD16A reporter stimulation (Figure 2A). As expected, BWZ reporters bearing the higher-affinity CD16A^V176^ allele demonstrated the same trend in timing as CD16A^F176^ reporters; however, CD16A^V176^ produced significantly higher responses and gave a signal in wells where no signal was detected by the low-affinity allelic variant (see 5- to 6-month time point; Figure 2A). We also addressed whether CD16A reporters could recognize antibodies cross-reacting to other SARS-CoV-2 spike variants (Delta B.1.617.2 strain and Omicron B.1.1.529 strain). Upon comparing Ancestral, Delta, and Omicron spike RBD in our assays, we observed similar trends with Delta and Ancestral Spike RBD; however, the signal was significantly lower with Delta using BWZ.CD16A^F176^ reporters (33% versus 14.1% at 2–3 months after second injection, and 86.5% versus 68.0% after booster). A similar pattern was observed with the BWZ.CD16A^V176^ reporters except stimulation was higher than the common allele reporters for all strains of virus, except for the Ancestral RBD after boost, where the stimulation by BWZ.CD16A^V176^ and BWZ.CD16A^F176^ were equally high (Figure 2, A and B). Interestingly, using Omicron Spike RBD, the BWZ.CD16A^F176^ reporters were not stimulated by plasma from donors following their second vaccination. Only antibodies generated after the booster were able to detect the Omicron spike RBD and stimulate the low-affinity reporters, whereas the high-affinity CD16A^V176^–expressing reporters could detect antibodies bound to Omicron Spike RBD at all time points following vaccination, albeit at lower levels than other Spike RBD strains (Figure 2C).

To determine whether antibodies generated in response to SARS-CoV-2 infection responded similarly in our assay, we obtained plasma from patients with COVID-19 from a previously described cohort in Seattle, Washington, USA (56, 57). Note that these patients were infected in 2020 and, therefore, were likely infected with the Ancestral SARS-CoV-2 strain. With our reporter assays, we tested plasma from patients with COVID-19 (*n* = 8) collected at acute (10–14 days following clinical diagnosis) and convalescent (2–3 months after initial onset of symptoms) time points. Our analysis of viremic patient plasma revealed that antibodies from the acute time point strongly stimulated BWZ.CD16A^F176^ reporters, and this signal decreased at the convalescent time point (Figure 2D). In addition, experiments using plates coated with Delta Spike RBD revealed a trend parallel to that with the ancestral Spike RBD (Figure 2E); however, with Omicron Spike RBD, the signal was only detected with some of the donor plasma at the acute time point using CD16A^F176^ reporters. Consistent with our previous observations, CD16A^V176^ reporter cells were able to detect the low amount of antibodies that cross-reacted with Omicron Spike RBD (Figure 2F). Therefore, these results demonstrate that the high-affinity CD16A allele can detect significantly lower amounts of antibodies and, thus, may be more protective during SARS-CoV-2 infection than the low-affinity common allele.

### Longitudinal clinical and immunologic profiling of patients with COVID-19 based on CD16A polymorphism.

**Figure 3:**
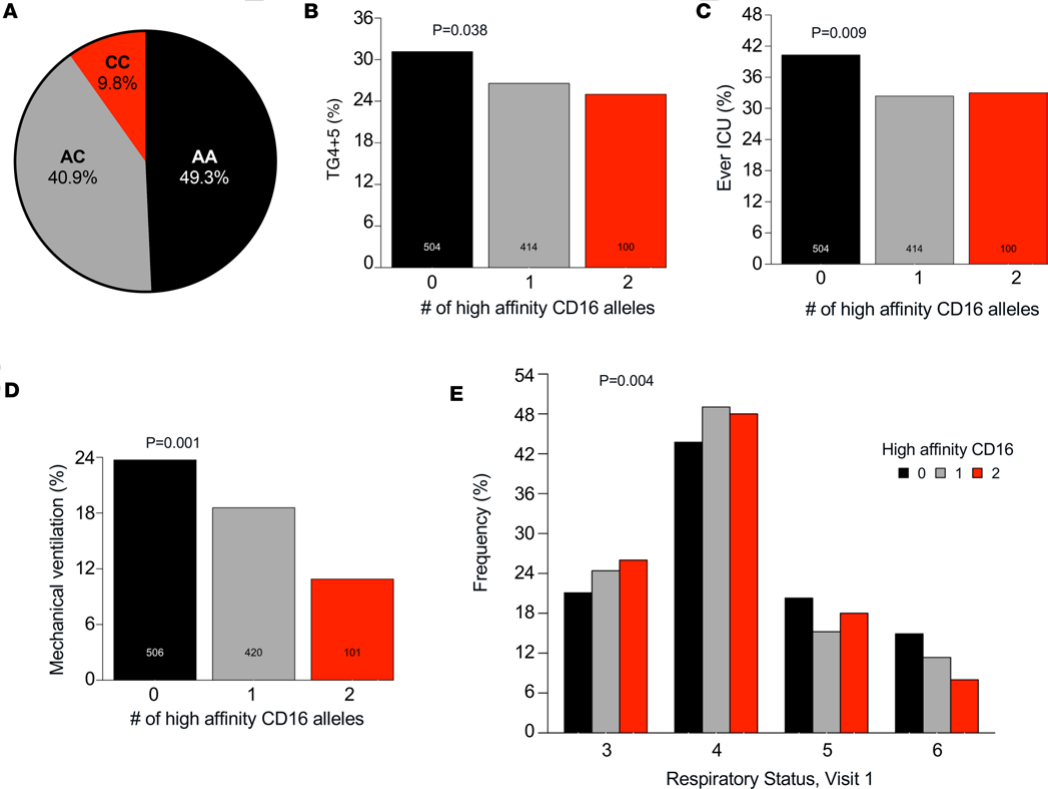
High-affinity CD16A^V176^ is associated with protection from severe COVID-19. (**A**) Breakdown of IMPACC participants by CD16A genotype, determined by Infinium Global Diversity Array sequencing of DNA. CC, homozygous for high affinity allele (*n* = 101); AC, heterozygous (*n* = 420); AA, homozygous for low affinity allele (*n* = 506). (**B**) Trajectory group 4 and 5 (severe COVID-19) status stratified by the number of high-affinity CD16A alleles present (odds ratio [OR] = 0.796, 95% CI = 0.641–0.985, adjusted *P* = 0.038). (**C**) ICU status among CD16A genotypes (OR = 0.767, 95% CI = 0.626–0.936, *P* = 0.009). (**D**) Percentage of IMPACC participants that were ever mechanically ventilated split by CD16A genotype (OR = 0.662, 95% CI = 0.515-0.845, *P* = 0.001). (**E**) Respiratory status breakdown by CD16A genotype at visit 1 (<48 hours from hospital admission), *P* = 0.004. 3 = hospitalized + no O_2_, 4 = hospitalized + required O_2_, 5 = noninvasive ventilation or high flow O_2_, and 6 = mechanical ventilation or ECMO. For all statistical tests unless otherwise stated, the high-affinity CD16A^V176^ variant allele count was treated as the independent variable and adjusted for age and sex. Analyses were modeled as clinical outcome-Y on CD16A^V176^ variant allele count-X by binomial regression for **B**–**D** and ordinal logistic regression for **E**.

To determine associations between the CD16A^V176^ allele and COVID-19 morbidity and mortality, we conducted a retrospective analysis of 1,027 patients within the IMPACC study who were hospitalized with SARS-CoV-2 infection between May 2020 to March 2021 (47, 48, 58). By Infinium Global Diversity Array sequencing of DNA from peripheral blood, we identified 101 (9.8%) participants who were homozygous for the high affinity allele (CC), 420 (40.9%) heterozygous (AC), and 506 (49.3%) who were homozygous for the low-affinity common allele (AA) (Figure 3A). In a public reference dataset of > 800,000 individuals sequenced (gnomAD), 17.6% of individuals are homozygous for the high-affinity CD16A^V176^ allele, whereas in our hospitalized IMPACC cohort, only 9.8% of patients carry this genotype. This lower frequency of the high-affinity allele in hospitalized individuals suggests an association with reduced disease severity.

We investigated several clinical and immunologic outcomes of interest stratified by CD16A genotype: anti–SARS-CoV-2 IgG titers, nasal viral load, COVID-19 severity, respiratory status, ICU admission, mechanical ventilation, and death. In a subset of patients, serum protein measurements (Olink, *n* = 948) and cytometry by time of flight (CyTOF, *n* = 788) of whole blood cells were performed. Median age, sex, and BMI were not significantly different across genotypes. The incidence of pulmonary disease (*P* = 0.033), chronic cardiac disease (*P* = 0.038), and smoking and/or vaping (*P* = 0.056) were slightly higher in those with the high-affinity allele. CD16A^V176^ was also found to be significantly enriched in individuals of African and European ancestry (*P* = 0.466 × 10^–6^) and less frequent in individuals of Hispanic or Latino ethnicity (*P* = 0.557 × 10^–8^). Additional details on participant demographics, comorbidities, and enrollment are in Tables 1 and 2. IMPACC collected biologic specimens and clinical data longitudinally, at specified visit dates (visit numbers) (47). Since the high-affinity CD16A polymorphism is relatively rare in the IMPACC patient cohort, we focused several of our analyses on visit 1 (<48 hours since hospital admission) and visit 4 (hospital day 14), which captures the most patient data recorded as well as the most dynamic window of acute SARS-CoV-2 infection. Visit 1 and visit 4 analyses were modeled as clinical outcome-Y on CD16A^V176^ variant allele count-X by linear (IgG AUC, viral rpM, protein concentration, cell type counts), binomial (disease severity, ICU status, mechanical ventilation, mortality), or ordinal logistic regression (respiratory status), as appropriate. All statistical tests were adjusted for age and sex.

### High-affinity CD16A^V176^ is associated with protection from severe COVID-19.

First, we evaluated how the high-affinity allele CD16A^V176^ affects COVID-19 severity. IMPACC assigned all participants to 1 of 5 COVID-19 trajectory groups (TGs) using latent class modeling based on a modified WHO score (58) within the first 28 days after admission. Patients in TG1–TG3 had relatively mild disease, whereas patients in TG4 and TG5 collectively had severe disease, including mortality. Upon assessment of the percentage of patients with severe COVID-19 (TG4+TG5) stratified by the number of high affinity alleles, CD16A^V176^ conferred 20.4% reduced odds of severe COVID-19 (CI = 0.641–0.985, *P* = 0.038) (Figure 3B). Furthermore, the CD16A^V176^ allele was associated with 23.3% reduced odds of ever going to the ICU (CI = 0.626–0.936, *P* = 0.009) (Figure 3C). Compared with study participants homozygous for the low-affinity common allele, those homozygous for CD16A^V176^ demonstrated 63.2% reduced cumulative odds of ever requiring mechanical ventilation (CI = 0.180–0.686, *P* = 0.001) (Figure 3D). We also investigated respiratory status, which was categorized according to the WHO criteria: 1 = not hospitalized + no limitations, 2 = not hospitalized + limitations, 3 = hospitalized + no O_2_, 4 = hospitalized + required O_2_, 5 = noninvasive ventilation or high flow O_2_, 6 = mechanical ventilation or ECMO, and 7 = death) (58). At visit 1, all patients were hospitalized, and none were deceased; thus, only scores 3–6 are represented. CD16A^V176^ was significantly associated with a lower WHO ordinal scale, meaning reduced clinical severity, at visit 1 (*P* = 0.004) (Figure 3E) and visit 4 (*P* = 0.025) (Supplemental Figure 2A). Despite the robust protection from severe COVID-19 associated with CD16A^V176^, we found no association between CD16A^V176^ alleles and mortality across the entire study (*P* = 0.788) (Supplemental Figure 2B). Taken together, these data demonstrate an association of the high-affinity CD16A allele with protection from the respiratory sequelae associated with a severe COVID-19 disease course, although it did not affect mortality in this sample population.

### High-affinity CD16A^V176^ is associated with lower anti–SARS-CoV-2 antibody titers.

**Figure 4:**
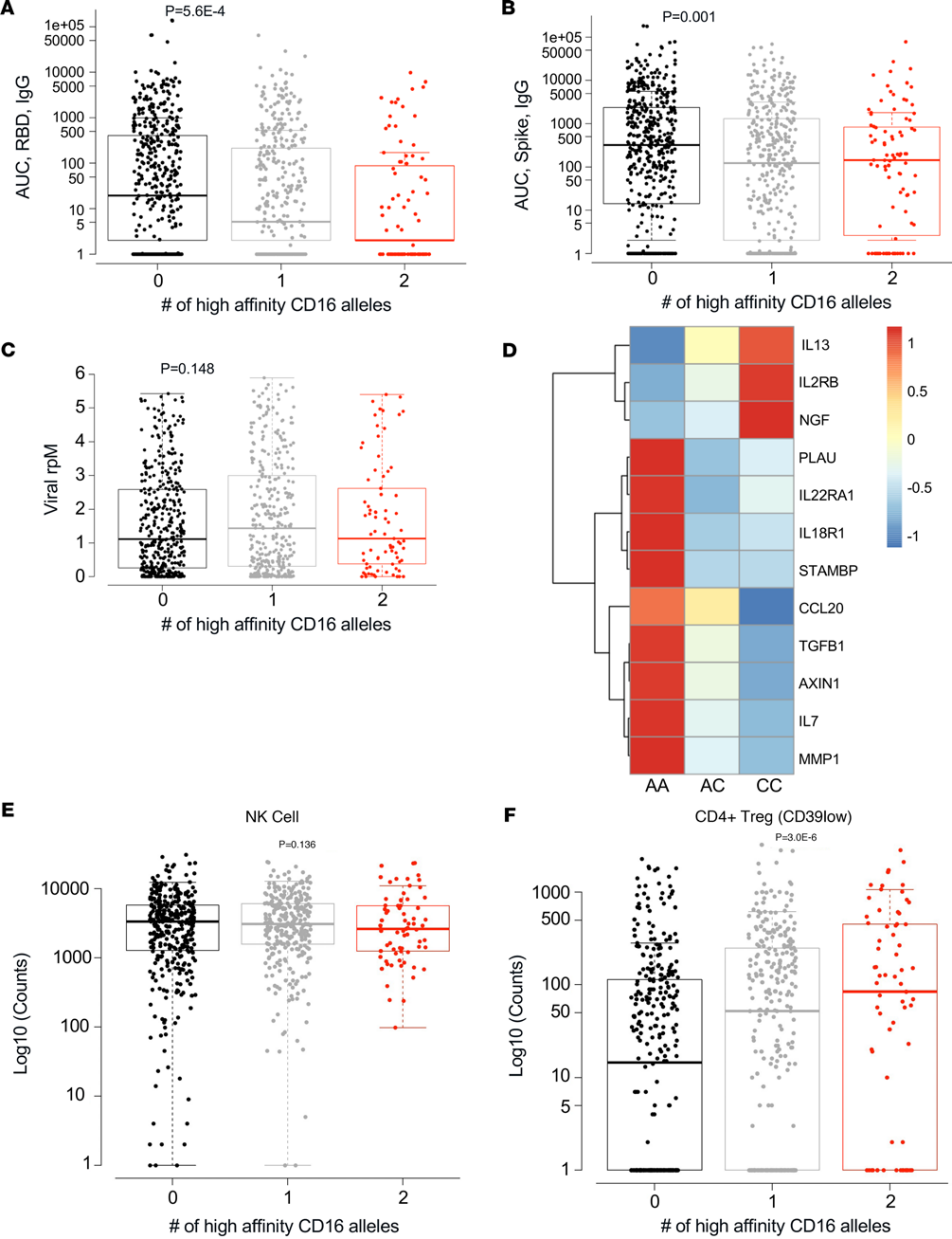
High-affinity CD16A^V176^ is associated with lower anti–SARS-CoV-2 antibody titers and globally reduced soluble mediators of inflammation. (**A**) Antibody levels against SARS-CoV-2 RBD were measured via ELISA as AUC and stratified by CD16A genotype (*P* = 5.6 × 10^–4^). (**B**) Antibody levels against SARS-CoV-2 spike were measured via ELISA and stratified by CD16A genotype (*P* = 0.001). (**C**) SARS-CoV-2 viral load reported as viral reads per million (rpM) was determined from nasal metagenomics and compared across CD16A genotypes (*P* = 0.148). Data from **A**–**C** are from cisit 1 (<48 hours from hospital admission). (**D**) Oligonucleotide-linked antibody detection (Olink) was performed on *n* = 948 participants to quantify cytokines, chemokines, and soluble receptors in serum. Displayed are the 12 that were significantly different across CD16A genotypes (*P* ≤ 0.05) after correcting for multiple testing. CC, homozygous high affinity. (**E** and **F**) Cytometry by time of flight (CyTOF) was performed on whole blood of *n* = 788 participants and immune cell counts were determined. NK cell counts (*P* = 0.136) in **E** and CD39^lo^CD4^+^ Treg counts (*P* = 3.0 × 10^–6^) in **F** were recorded split by CD16A genotype. Analyses were modeled as clinical outcome-Y on CD16A^V176^ variant allele count-X by linear regression.

The high-affinity CD16A^V176^ allele was associated with reduced anti–SARS-CoV-2 RBD IgG titers and anti-spike titers at visit 1 (*P* = 5.6 × 10^–4^, *P* = 0.001) (Figure 4, A and B) and visit 4 (*P* = 0.01, *P* = 0.006) (Supplemental Figure 2, C and D). Interestingly, there was no difference found in SARS-CoV-2 viral load (reported as SARS-CoV-2 reads per million [rpM] from nasal metagenomics) upon comparing CD16A genotypes at visit 1 (*P* = 0.148) (Figure 4C) or visit 4 (*P* = .246) (Supplemental Figure 2E). In addition, the correlation between anti–SARS-CoV-2 antibody titers and viral load was not different by genotype (Supplemental Figure 3, A and B). All clinical outcomes discussed are detailed in Table 3.

### Participants homozygous for CD16A^V176^ have globally reduced soluble mediators of inflammation.

Since the CD16A^V176^ appears protective from severe COVID-19 and respiratory burden, we assessed the link between protein markers and the CD16A polymorphism. IMPACC has previously quantified cytokines, chemokines, and soluble receptors in patient serum via oligonucleotide-linked antibody detection (Olink). Diray-Arce et al. (48) used the ImmuneXpresso (59) to group these soluble proteins into 7 Olink modules based on their action on immune cells and found Module 3 (labeled “activators of NK cells”) was higher in participants with mild disease, while Modules 1, 2, 4, and 6 were higher in participants with severe disease. Out of the 92 soluble proteins measured, there were 12 significant differences after correcting for multiple testing in plasma proteins based on CD16A polymorphism (Figure 4D). Nine proteins were significantly down and 3 were significantly up in the participants homozygous for CD16A^V176^. The majority of proteins that were downregulated are in modules previously associated with severe disease, including PLAU, STAMBP, and AXIN1 (Table 4), while the 3 proteins that were upregulated are in Module 0, which was not associated with clinical outcomes in the prior study. Two plasma proteins significantly downregulated in the high-affinity individuals, IL-18R1 and CCL20, are in Module 2 (labeled “proinflammatory, produced by monocytes”), which was most significantly associated with the most severe group, TG5. IFN-γ trended higher in participants homozygous for low-affinity CD16A, but it was not significant (data not shown). Overall, these Olink data reveal that, at a broad level, participants homozygous for the high-affinity CD16A^V176^ have lower levels of soluble mediators of inflammation in the periphery compared with participants homozygous for the common low-affinity allele.

### No difference in NK cell frequencies based on CD16A genotype.

To determine whether the CD16A polymorphism is associated with global changes in the number of circulating immune cells, we examined immune cell frequencies in CYTOF data from patients’ whole blood, beginning with the frequencies of NK cells with or without the CD16A polymorphism. Total NK cell counts, as well as NK cell subsets (mature CD56^lo^CD16^hi^, immature CD56^hi^CD16^lo^) were not different across CD16A genotypes (Figure 4E and Supplemental Figure 4, A and B). CD16A can also be expressed on monocytes and neutrophils, so we assessed the relationship between their frequencies and CD16A^V176^. Monocyte frequencies were slightly higher in homozygous CD16A high-affinity participants (*P* = 0.034), but there was no difference in neutrophil frequencies (*P* = 0.618) (Supplemental Figure 4, C and D). Finally, we looked at other immune cell populations that do not express CD16A to assess whether they are indirectly affected by the CD16A polymorphism. Interestingly, there was a striking finding of significantly increased frequencies of CD39^lo^CD4^+^ Tregs in the participants homozygous for CD16A^V176^ (*P* = 3 × 10^–6^) with no difference in CD39^hi^CD4^+^ Tregs (*P* = 0.192) (Figure 4F and Supplemental Figure 4E). Of note, CD39^hi^ Tregs have been found to be associated with severe COVID-19, and CD39 upregulation has been recorded in other viral illnesses (60).

## Discussion

Although NK receptor CD16A has been thoroughly studied in the context of cancer, where it serves as an attractive target for antitumor therapies (2, 3), the importance of CD16A function in COVID-19 is much less clear. It remains uncertain whether NK cell–mediated ADCC limits SARS-CoV-2 spread and disease severity or contributes to the immunopathogenesis of severe COVID-19. We hypothesized that perhaps ADCC is a double-edged sword that can either help or hurt the host depending on factors such as CD16A allelic variants. Here, we created an in vitro system to study the Fc effector functionality of common allele CD16A^F176^ versus the high-affinity minor allele CD16A^V176^ in response to anti–SARS-CoV-2 antibodies. In these assays, CD16A^V176^ reporters were more sensitive and robust activators than CD16A^F176^ reporters. This was evident in plasma from vaccinated individuals or from patients infected with SARS-CoV-2. It is important to note that both CD16A reporters demonstrated the strongest stimulation in assays with Ancestral spike protein and this is likely due to the initial COVID-19 vaccinations being made specifically against the Ancestral spike variant. Strikingly, the high-affinity CD16A reporters could still detect the low amounts of antibodies developed against SARS-CoV-2 Ancestral spike RBD that cross-reacted with Omicron Spike RBD.

To investigate associations between CD16A genotype and clinical outcomes, we conducted an analysis on 1,027 hospitalized patients with COVID-19 from the IMPACC study, a uniquely rich longitudinal dataset with extensive transcriptomic, proteomic, and clinical data. While Vietzen et al. (38) reported CD16A^V176^ was associated with severe COVID-19 in their limited cohort of 197 Austrian participants, our larger cohort showed the opposite. In the IMPACC cohort, the CD16A^V176^ allele was associated with reduced risk of mechanical ventilation, ICU admission, respiratory severity, and severe COVID-19 trajectories. These results may reflect the diverse patient demographics (Tables 1 and 2) in our cohort, suggesting that the CD16A^V176^ allele confers protection from a severe disease course and the associated respiratory sequelae. It is important to note, however, that while CD16A^V176^ was associated with protection from the severe COVID-19 trajectory course, no differences were found in mortality upon stratification by CD16A genotype. Interestingly, we found slightly higher rates of pulmonary disease (*P* = 0.033), chronic cardiac disease (*P* = 0.038), and smoking/vaping (*P* = 0.056) among individuals with the high-affinity allele. Despite these comorbidities, these individuals still had reduced risk of severe COVID-19, suggesting that the observed protective effect may not be merely a result of lower baseline health risk.

Degranulation assays reported by several research groups demonstrate that sera from hospitalized COVID-19 individuals induced more robust ADCC than sera from those with milder, self-limiting disease (38, 39). Stronger ADCC responses in hospitalized patients could be due to prolonged antigen exposure due to higher viral loads. Interestingly, ADCC bioassays with sera from patients with severe COVID-19 demonstrate more activity from those that survived versus those that died, peaking around 11 days after disease onset (32). When taken together with our in vitro findings, this suggests that the CD16A^V176^ polymorphism in hospitalized patients may enable NK cells to execute more timely and/or potent ADCC upon infection with SARS-CoV-2 to limit severe manifestations. Perhaps in these hospitalized patients, an efficacious ADCC response to SARS-CoV-2 infection requires strong, early effector function, which high-affinity CD16A^V176^ executes. Fc effector function by the common low-affinity allele is weaker and delayed due to the lack of sensitivity to antibody titer, thus it may fail to limit viral control and instead contributes to the inflammatory immunopathogenesis.

In the IMPACC dataset, there is a strong negative correlation between viral load and anti–SARS-CoV-2 antibody titers. One would expect that because the participants homozygous for CD16A^V176^ have lower anti–SARS-CoV-2 titers, they would have a higher viral load and be more clinically ill; however, that was not the case. Since viral load is overall strongly positively correlated with disease severity in the IMPACC cohort (48) and other cohorts (61, 62), we hypothesize that the high-affinity allele CD16A^V176^ may weaken the relationship between viral load and clinical severity. Indeed, upon comparing viral loads in TG1–TG3 (mild disease) versus TG4 and TG5 (severe disease), participants homozygous for the low-affinity allele and heterozygotes have viral loads trending upward in TG4 and TG5, while participants homozygous for the high-affinity allele had viral loads trending downward. Further work needs to be done to understand why high-affinity CD16A^V176^ is associated with significantly lower anti–SARS-CoV-2 antibody titers.

While the results here suggest the CD16A polymorphism plays a role in shaping COVID-19 clinical severity, the exact role of ADCC and whether it functions in a protective or pathogenic manner may be pathogen dependent or related to outside host factors. Antibody glycosylation patterns have emerged as a key determinant in host immune responses to enveloped viruses, such HIV and COVID-19. Severely ill patients with COVID-19 were found to have higher levels of afucosylated anti–SARS-CoV-2 IgG1 antibodies (which lack a core fucosylation on the Fc region) than those with asymptomatic or mild self-limited infection (63). Several groups have demonstrated that afucosylated IgG variants have increased CD16A affinity (64, 65), which is commonly used in glyco-engineered monoclonal antitumor antibody generation (66).

The persistence of the low-affinity CD16A variant suggests evolutionary trade-offs that balance immune defense and regulation. While the high-affinity variant may enhance ADCC and pathogen clearance, it could also lead to excessive immune activation and inflammation. In autoimmune-prone individuals, a lower-affinity receptor may serve a protective role by reducing unnecessary immune activation. Additionally, CD16A exhibits extensive polymorphism, indicating it could be a target of pathogen-driven evolution. Such diversity suggests ongoing selective pressures, where hosts may evolve CD16A variants to evade pathogen exploitation, maintaining multiple functionally distinct alleles in the population.

Our study has several limitations. The power of our analysis of the IMPACC cohort was limited by the rare frequency homozygotes for the CD16A high-affinity minor allele in the participant population (9.8%). Although plasma taken from individuals acutely infected with SARS-CoV-2 and vaccinated for COVID-19 is a helpful proxy in our in vitro CD16A reporter assays to assess Fc effector function, ideally, ADCC capacities should be assessed with primary NK cells collected from patients with SARS-CoV-2 across the severity spectrum. While measuring ADCC in NK cells from patients with COVID-19 was out of the scope of this paper, our findings suggest individuals carrying the CD16A^V176^ allele have enhanced NK cell effector functions, leading to reduced inflammatory markers and protection against severe COVID-19. These results parallel findings in the HIV and cancer literature in which the high-affinity CD16A^V176^ correlates to enhanced ADCC activity in HIV elite controllers and improved responses to rituximab in lymphoma patients. The overlap between these contexts underscores the importance of CD16A-mediated ADCC in both antiviral immunity and therapeutic antibody responses. Future studies should explore whether the protective effects observed in our COVID-19 cohort extend to enhanced ADCC-driven viral clearance, similar to HIV elite controllers, and whether CD16A^V176^ influences responses to other monoclonal antibody-based therapies.

In conclusion, our study demonstrated that the high-affinity CD16A allele has a greater capacity to detect antibodies generated to vaccination and to acute infection. This allele may be more protective against Spike RBD variants; thus, individuals carrying the high affinity CD16A allele may be less likely to develop severe respiratory manifestations of COVID-19.

## Methods

### Sex as a biological variable.

Our study cohort examined 405 women (39%) and 622 men (61%). Female sex was not significantly associated with CD16A polymorphism. Sex and age were used as covariates in statistical testing.

### Clinical study design.

IMPACC is an observational longitudinal study on hospitalized patients with COVID-19 (47). Participants were enrolled from 20 hospitals affiliated with 15 academic institutions geographically distributed across the United States, totaling 1,164 patients hospitalized with symptoms or signs of COVID-19 between May 5, 2020, and March 19, 2021. Eligible participants include patients hospitalized with symptoms of SARS-CoV-2 infection confirmed by reverse transcription PCR (RT-PCR). The comprehensive study design, the schedule for collecting clinical data and biological samples, and the participants’ demographic details have been previously reported (47, 48, 58). Detailed information on patient demographics and study enrollment are listed in Tables 1 and 2. Clinical assessments and patient samples (e.g., nasal swabs, blood, endotracheal aspirates) were collected within 48 hours of hospitalization (visit 1) and on days 4, 7, 14, 21, and 28 after hospital admission. If a patient was discharged before day 14 or 28, attempts were made to collect clinical data and samples at days 14 and 28 in an outpatient setting. The severity of the disease was evaluated using a 7-point ordinal scale based on the degree of respiratory illness, and longitudinal dynamics of disease severity were clustered to define 5 TGs.

### Clinical outcome variables.

Longitudinal measures of the WHO 7-point severity ordinal scale over time were clustered into 5 TGs using group-based trajectory modeling, a likelihood-based approach commonly used to group time series of clinical data, as described previously (58). For the severity analysis, we defined mild participants as those with TG1–TG3, and severe participants as those with TG4–TG5, with TG5 representing all fatal cases within 28 days of admission. Mechanical ventilation was defined as a respiratory ordinal score of 6. Additionally, we compared patients with confirmed mortality at any time during the study or within 28 days of hospital admission against survivors.

### IMPACC cohort genotyping.

DNA was extracted, and samples were genotyped on the Illumina Global Diversity Array as previously described. For this analysis, we extracted the CD16A rs396991 polymorphism.

### BWZ reporter cell generation.

BWZ cells were provided by N. Shastri (UC Berkeley) (52). HEK293T cells were obtained from the American Type and Culture Collection (ATCC). Cells were cultured in complete RPMI-1640 (RPMI) or DMEM-HG supplemented with 2 mM glutamine, 100 U/mL penicillin, 100 μg/mL streptomycin, 50 μg/mL gentamicin, 110 μg/mL sodium pyruvate, 50 μM 2-mercaptoethanol, 10 mM HEPES, and 10% FBS. PBMCs were isolated from blood using Ficoll-Plaque PLUS and cultured overnight in supplemented RPMI-1640 media containing 10% FBS and 200 U/mL of recombinant human IL-2 (Teceleukin). Gene fragments containing the extracellular domains of human CD16A (*FCGR3A*) were designed with flanking 5′ XhoI and 3′ NotI restriction enzyme cut sites and ordered from Integrated DNA Technologies. Gene fragments encoding the lower affinity (CD16A^F176^) as well as the higher affinity (CD16A^V176^) allele were included. These constructs were subcloned into pMSCV2.2-IRES-EGFP vector expressing a type I fusion cassette containing a mouse CD8α transmembrane domain and a mouse CD3ζ intracellular domain to yield CD3ζ-CD16A^F176^ and CD3ζ-CD16A^V176^ chimeric receptors that were used to generate reporters, as previously described (52). All vectors were sequenced for validation of desired inserts. For transfections, HEK293T cells were plated one day prior to transfection in 6-well plates (6 × 10^5^/well). Transfections were performed using Lipofectamine 2000 according to the manufacturer’s protocol (Thermo Fisher Scientific). Retroviruses were generated by cotransfecting pMSCV2.2-IRES-EGFP vectors into HEK293T along with packaging plasmids, and viral supernatant was then used to transduce BWZ cells. Cells were then sorted for expression of EGFP prior to flow cytometric sorting for CD16A cell surface expression.

### BWZ reporter assays.

Plate-bound stimulations were conducted using high-binding EIA/RIA plates (Corning) precoated with purified antibodies (10–30 μg/mL in PBS) overnight. The next day, wells were thoroughly washed with PBS, and then BWZ reporters (5 × 10^4^) were added to the wells and plates were incubated overnight. Stimulations using 30.2 nM PMA and 0.5 μM ionomycin (Iono) served as positive controls, while media alone served as negative controls. The following day, cells were pelleted by centrifugation (700*g* for 3 minutes), washed with PBS, and resuspended in 150 μL of CPRG buffer (90 mg/L chlorophenol-red-b-D-galactopyranoside [Roche], 9 mM MgCl_2_, 0.1% NP-40 in PBS), and assays were developed at room temperature. Readings were recorded using a microplate reader (Tecan Life Sciences) at OD 595–655 nm. Data were normalized to control values using the following formula: % receptor specific stimulation = (treatment – negative control)/(positive control – negative control) × 100%. For detection of anti-RBD IgGs in plasma, high-binding EIA/RIA plates (Corning) were coated overnight with neutravidin (10 μg/mL in PBS), washed thoroughly, incubated for biotinylated Spike RBD protein (5 μL/mL in PBS) for 2 hours at room temperature, washed thoroughly, and blocked using 200 μL of SuperBlock solution (Thermo Fisher Scientific) for 1 hour at room temperature; then, heat inactivated plasma was added at different dilutions for 2 hours at room temperature. After incubation, wells were thoroughly washed, and BWZ reporter cells were added (5 × 10^4^/well) and incubated overnight. The following day, assays were developed as described above. Biotinylated SARS-CoV-2 spike RBD proteins (Wuhan and Delta strain B.1.617.2) were generated as previously described (55). Biotinylated SARS-CoV-2 Omicron spike RBD (strain B.1.1.529) were purchased from R&D Systems. Data were normalized to control values using the following formula: % receptor specific stimulation = (treatment – negative control)/(positive control – negative control) × 100%.

### Stimulation of primary NK cells.

PBMCs were incubated overnight in 200 U/mL rhIL-2 and then used as effectors in plate-bound stimulation assays. Briefly, Spike RBD was anchored to high-binding chemistry EIA/RIA plates (Corning) using neutravidin and coated with plasma from vaccinated donors or patients with COVID-19, and then PBMCs were layered onto wells to determine NK cell activation. PBMCs were incubated for 5 hours in the presence of anti-CD107A antibody and monensin (1 μL anti-CD107a mAb/well and 2 μM monensin) (BioLegend, H4A3). Stimulations using 30.2 nM PMA and 0.5 μM Iono (PMA+Iono) served as positive controls, while media alone served as negative controls. After incubations, cells were stained for surface markers and live/dead, fixed, permeabilized, stained for intracellular IFN-γ, and then analyzed using flow cytometry. To control for patient variability, we normalized the activation of the conditions relative to the control of the experiment using the following calculation: Norm. % activation = (activation by condition – activation in negative control)/(activation by positive control – activation by negative control).

### Patient plasma and PBMCs for CD16A reporter and primary NK cell assays.

For the CD16A reporter and primary NK cell assays, vaccinated plasma samples were obtained using protocols approved by the IRBs of the UCSF (IRB study no. 1000265). Prevaccinated blood was collected from healthy volunteers before the COVID-19 pandemic, and then the same healthy volunteers donated blood 2–3 months after the second vaccine, 5–6 months after the second vaccine, and 1–4 weeks after the booster. All vaccines were Pfizer-BioNTech or Moderna. The second vaccine was administered to healthy volunteers between February and April of 2021, and the booster was administered between November and December of 2021. Viremic plasma samples were obtained from the INCOV cohort, as previously described (56, 57) and came from patients with SARS-CoV-2 identified at 5 hospitals within the Swedish Medical Center network and affiliated clinics in the Puget Sound region near Seattle, Washington, USA. All participants provided written, in-person informed consent prior to enrollment. Blood samples were collected and analyzed at 2 time points: a few days after the initial clinical diagnosis, when patients were primarily in the acute stage of infection (acute), and 2–3 months following the onset of symptoms (convalescent). The study protocol was approved by the IRB of Providence St. Joseph Health (IRB study no. STUDY2020000175) and the Western Institutional Review Board (WIRB, IRB study no. 20170658).

### Flow cytometry.

Cells were stained with primary mAbs in FACS buffer (2.5% FBS, 1 mM EDTA, and 0.03% NaN3 in PBS) on ice for 25 minutes, washed, incubated with secondary antibodies for another 25 minutes, and then analyzed using a LSRII conventional flow cytometer (BD Biosciences). For functional assessment of primary NK cells, cells were fixed for 30 minutes using FluroFix (BioLegend) prior to staining intracellularly according to manufacturer’s instruction (BioLegend). Cells were gated by forward and side light scatter properties, and then for cell viability using propidium iodide (PI) or Zombie Red (BioLegend) exclusion. Data were analyzed using FlowJo software (FlowJo). Anti–human CD16 (3G8 or B73.1, BioLegend), –human CD107A (H4A3, BioLegend), –human IFN-γ (4S.B3, BioLegend), -CD56 (HCD56, BioLegend), -CD19 (HIB19, BioLegend), -CD14 (63D3, BioLegend), -NKp30 (P30-15, BioLegend), -CD57 (QA17A04, BioLegend), -NKG2C (134591, R&D Systems), -NKp80 (5D12, BioLegend), –mouse IgG1 isotype-matched control (MOPC-21, BioLegend), –mouse IgG2a isotype-matched control (MOPC-173, BioLegend), –rat IgG2a isotype-matched control (RTK2758, BioLegend), –human isotype IgG1 (BioXCell), –human isotype IgG2 (BioXCell), –human isotype IgG3 (Novus Biologicals), and –human isotype IgG4 (BioLegend). All secondary PE- or APC-conjugated streptavidin reagents were purchased from BioLegend.

### IMPACC antibody measurements.

Antibody levels against the recombinant SARS-CoV-2 Spike protein were measured in the blood using a research-grade ELISA as described (48). The OD was measured and the AUC was calculated. SARS-CoV-2 viral abundance was calculated as log_10_ (rpM+1), where rpM is the rpM of SARS-CoV-2 as measured by nasal meta transcriptomics.

### Analysis of serum inflammatory protein (Olink) data.

All samples were processed with the Olink multiplex assay inflammatory panels (Olink Proteomics), according to the manufacturer’s instructions and as previously described (48). This inflammatory panel included 92 proteins associated with human inflammatory conditions. Target protein quantification was performed by real-time microfluidic quantitative PCR (qPCR) via the Normalized Protein Expression (NPX) manager software. Data were normalized using internal controls in every sample, interplate control and negative controls, and correction factor and expressed as log_2_ scale proportional to the protein concentration. For additional quality control, we set any NPX measurements below the assay’s limit of detection (LOD) to zero.

### Analysis of CyTOF data.

IMPACC PBMCs were phenotyped on the Fluidigm Helios mass cytometer using distinct panels of surface and intracellular markers, and the cell types were annotated using an automated annotation pipeline as previously described (48). Prior to analysis, cells identified as RBCs, multiplets, and debris, and those that were not identifiable with high confidence were removed. These counts were converted to proportions per sample, by dividing each cell type count by the total cell count.

### Statistics.

All in vitro data were analyzed using Prism 9 (GraphPad), employing 2-way ANOVA analysis or 2-tailed Student’s *t* test. Data are shown as mean ± SEM or mean ± SD. All data are representative of at least 2 independent experiments or 3–4 biological replicates. For all IMPACC analyses, unless otherwise stated, the CD16A^V176^ variant allele count was treated as the independent variable. Visit 1 and visit 4 analyses were modeled as clinical outcome-Y on CD16A^V176^ variant allele count by binomial or ordinal logistic regression, as appropriate. Models were considered significant for *P* < 0.05. All analyses were performed unadjusted and adjusted for age and sex.

### Study approval.

NIAID staff conferred with the Department of Health and Human Services Office for Human Research Protections (OHRP) regarding the potential applicability of the public health surveillance exception (45CFR46.102) to the IMPACC study protocol. The OHRP concurred that the study satisfied the criteria for the public health surveillance exception, and the IMPACC study team sent the study protocol and the participant information sheet for review and assessment to the IRBs at the participating institutions. Twelve institutions elected to conduct the study as public health surveillance, whereas 3 sites with prior IRB-approved biobanking protocols elected to integrate and conduct the IMPACC study under their institutional protocols (The University of Texas at Austin, Austin, Texas, USA; IRB 2020-04-0117; UCSF, San Francisco, California, USA; IRB 20-30497; Case Western Reserve University, Cleveland, Ohio, USA; IRB STUDY20200573) with informed consent requirements. In addition, approved IRB protocols at UCSF (IRB study number 1000265), at Providence St. Joseph Health (IRB study no. STUDY2020000175), and the Western Institutional Review Board (WIRB, IRB study no. 20170658) were used for collection of additional samples. Participants enrolled under the public health surveillance exclusion were provided information sheets describing the study, samples to be collected, and plans for data deidentification and use. Those who opted not to participate after reviewing the information sheet were not enrolled. In addition, participants did not receive compensation for study participation while they were inpatients and were subsequently offered compensation during outpatient follow-ups.

### Data availability.

Data files are available at ImmPort under accession no. SDY1760 and dbGAP accession no. phs002686.v1.p1. Data values for all graphs in this paper are available in the Supporting Data Values file.

## Author contributions

AEQ, OAA, and LLL designed the research, AEQ, OAA, and TT performed the experiments, the IMPACC Network (EC, DD, HTM, SKS, RRM, F Krammer, CRL, OL, LRB, EM, LIRE, GAM, RPS, CBC, EKH, ACS, DAH, DBC, F Kheradmand, MAA, SCB, NIAH, JPM, CLH, WBM, B Pulendran, KCN, MMD, AFS, VS, MK, CB, CSC, DJE, JS, AO, B Peters, SHK, ADA, JDA, PMB, NR) designed the clinical analysis and/or acquired the clinical data. IL, SAL, and JAW provided in vitro reagents. YS, JRH, and JDG provided clinical samples. HP analyzed and performed statistics on the clinical data, AEQ, HP, OAA, DRC, EFR and LLL contributed to clinical data interpretation, AEQ, OAA, TT, and LLL contributed to in vitro data analysis and interpretation. AEQ, HP, and OAA wrote the manuscript. OAA, HP, EFR, and LLL critically reviewed and approved the final version of the manuscript.

## Supplementary Material

Supplemental data

Supporting data values

## Figures and Tables

**Table 1:**
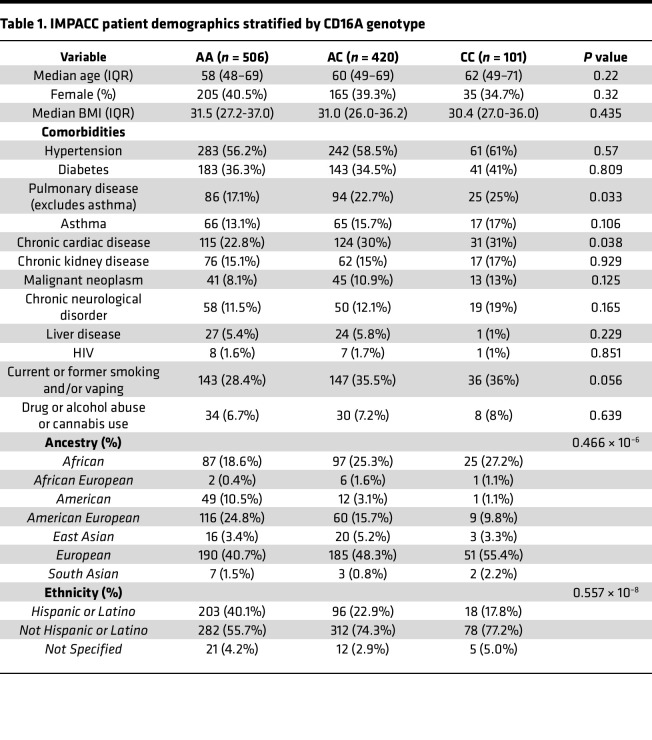
IMPACC patient demographics stratified by CD16A genotype

**Table 2:**
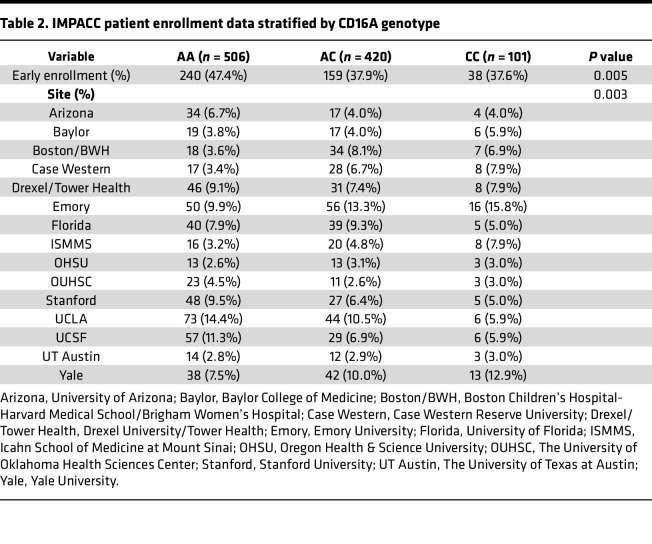
IMPACC patient enrollment data stratified by CD16A genotype

**Table 3:**
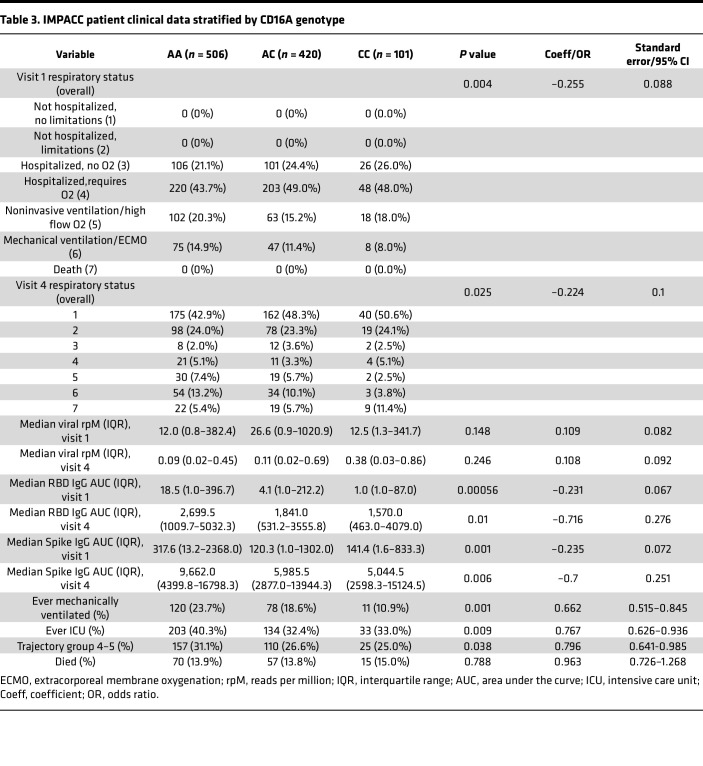
IMPACC patient clinical data stratified by CD16A genotype

**Table 4:**
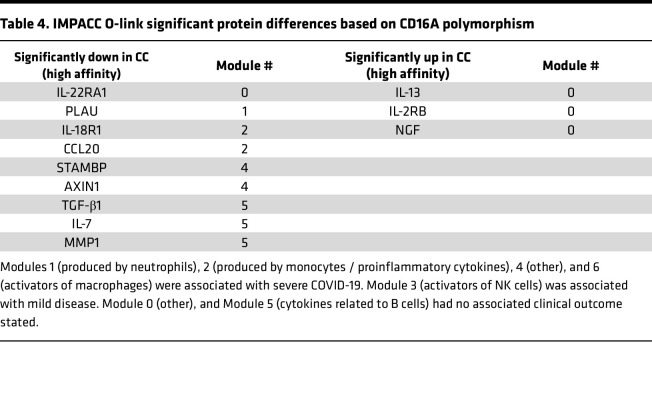
IMPACC O-link significant protein differences based on CD16A polymorphism

## Version 1 05/22/2025

In-Press Preview

## Version 2 07/08/2025

Electronic publication
