# Supplementary material for: High-affinity CD16A polymorphism associated with reduced risk ofsevere COVID-19
Source: JCI Insight. 2025 May 22;10(13):e191314. doi: 10.1172/jci.insight.191314 (PMC12306582; doi:10.1172/jci.insight.191314)
Supplement: Supplemental data [file jciinsight-10-191314-s160.pdf]

Supplemental Acknowledgements:

IMPACC Network

National Institute of Allergy and Infectious Diseases, National Institute of Health, Bethesda, MD 20814, USA: Patrice M. Becker, Alison D. Augustine, Steven M. Holland, Lindsey B. Rosen, Serena Lee, Tatyana Vaysman

Clinical and Data Coordinating Center (CDCC), Precision Vaccines Program, Boston Children's Hospital, Harvard Medical School, Boston, MA 02115, USA: Al Ozonoff, Joann Diray-Arce, Jing Chen, Alvin T. Kho, Carly E. Milliren, Annmarie Hoch, Ana C. Chang, Kerry McEnaney, Caitlin Syphurs, Brenda Barton, Claudia Lentucci, Maimouna D. Murphy, Mehmet Saluvan, Tanzia Shaheen, Shanshan Liu, Marisa Albert, Arash Nemati Hayati, Robert Bryant, James Abraham, Mitchell Cooney, Meagan Karoly

Benaroya Research Institute, University of Washington, Seattle, WA 98101, USA: Matthew C. Altman, Naresh Doni Jayavelu, Scott Presnell, Bernard Kohr, Tomasz Jancsyk, Azlann Arnett

La Jolla Institute for Immunology, La Jolla, CA 92037, USA: Bjoern Peters, James A. Overton, Randi Vita, Kerstin Westendorf

Knocean Inc. Toronto, ON M6P 2T3, Canada: James A. Overton

Precision Vaccines Program, Boston Children's Hospital, Harvard Medical School, Boston, MA 02115, USA: Ofer Levy, Hanno Steen, Patrick van Zalm, Benoit Fatou, Kinga K. Smolen, Arthur Viode, Simon van Haren, Meenakshi Jha, David Stevenson, Athena N. Nguyen, Alec L. Plotkin, Sanya Thomas, Boryana Petrova, Naama Kanarek

Brigham and Women's Hospital, Harvard Medical School, Boston, MA 02115, USA: Lindsey R. Baden, Kevin Mendez, Jessica Lasky-Su, Alexandra Tong, Rebecca Rooks, Michael Desjardins, Amy C. Sherman, Stephen R. Walsh, Xhoi Mitre, Jessica Cauley, Xiaofang Li, Bethany Evans, Christina Montesano, Jose Humberto Licona, Jonathan Krauss, Nicholas C. Issa, Jun Bai Park Chang, Natalie Izaguirre

Metabolon Inc, Morrisville, NC 27560, USA: Scott R. Hutton, Greg Michelotti, Kari Wong

Prevention of Organ Failure (PROOF) Centre of Excellence, University of British Columbia, Vancouver, BC V6T 1Z3, Canada: Scott J. Tebbutt, Casey P. Shannon

Case Western Reserve University and University Hospitals of Cleveland, Cleveland, OH 44106, USA: Rafick-Pierre Sekaly, Slim Fourati, Grace A. McComsey, Paul Harris, Scott Sieg, George Yendewa, Mary Consolo, Heather Tribout, Susan Pereira Ribeiro

Drexel University, Tower Health Hospital, Philadelphia, PA 19104, USA: Charles B. Cairns, Elias K. Haddad, Michele A. Kutzler, Mariana Bernui, Gina Cusimano, Jennifer Connors, Kyra Woloszczuk, David Joyner, Carolyn Edwards, Edward Lee, Edward Lin, Nataliya Melnyk, Debra L. Powell, James N. Kim, I. Michael Goonewardene, Brent Simmons, Cecilia M. Smith, Mark Martens, Brett Croen, Nicholas C. Semenza, Mathew R. Bell, Sara Furukawa, Renee McLin, George P. Tegos, Brandon Rogowski, Nathan Mege, Kristen Ulring, Pam Schearer, Judie Sheidy, Crystal Nagle

MyOwnMed Inc., Bethesda, MD 20817, USA: Vicki Seyfert-Margolis

Emory School of Medicine, Atlanta, GA 30322, USA: Nadine Rouphael, Steven E. Bosinger, Arun K. Boddapati, Greg K. Tharp, Kathryn L. Pellegrini, Brandi Johnson, Bernadine Panganiban, Christopher Huerta, Evan J. Anderson, Hady Samaha, Jonathan E. Sevransky, Laurel Bristow, Elizabeth Beagle, David Cowan, Sydney Hamilton, Thomas Hodder, Amer Bechnak, Andrew Cheng, Aneesh Mehta, Caroline R. Ciric, Christine Spainhour, Erin Carter, Erin M. Scherer, Jacob Usher, Kieffer Hellmeister, Laila Hussaini, Lauren Hewitt, Nina Mcnair, Susan Pereira Ribeiro, Sonia Wimalasena

Icahn School of Medicine at Mount Sinai, New York, NY 10029, USA: Ana Fernandez-Sesma, Viviana Simon, Florian Krammer, Harm van Bakel, Seunghee Kim-Schulze, Ana Silvia Gonzalez-Reiche, Jingjing Qi, Brian Lee, Juan Manuel Carreño, Gagandeep Singh, Ariel Raskin, Johnstone Tcheou, Zain Khalil, Adriana van de Guchte, Keith Farrugia, Zenab Khan, Geoffrey Kelly, Komal Srivastava, Lily Q. Eaker, Maria C. Bermúdez-González, Lubbertus C.F. Mulder, Katherine F. Beach, Miti Saksena, Deena Altman, Erna Kojic, Levy A. Sominsky, Arman Azad, Dominika Bielak, Hisaaki Kawabata, Temima Yellin, Miriam Fried, Leeba Sullivan, Sara Morris, Giulio Kleiner, Daniel Stadlbauer, Jayeeta Dutta, Hui Xie, Manishkumar Patel, Kai Nie, Brian Monahan

Immunai Inc., New York, NY 10016, USA: Adeeb Rahman

Oregon Health & Science University, Portland, OR 97239, USA: William B. Messer, Catherine L. Hough, Sarah A.R. Siegel, Peter E. Sullivan, Zhengchun Lu, Amanda E. Brunton, Matthew Strand, Zoe L. Lyski, Felicity J. Coulter, Courtney Micheletti

Stanford University School of Medicine, Palo Alto, CA 94305, USA: Holden Maecker, Bali Pulendran, Kari C. Nadeau, Yael Rosenberg-Hasson, Michael Leipold, Natalia Sigal, Angela Rogers, Andrea Fernandes, Monali Manohar, Evan Do, Iris Chang, Alexandra S. Lee, Catherine Blish, Henna Naz Din, Jonasel Roque, Linda N. Geng, Maja Artandi, Mark M. Davis, Neera Ahuja, Samuel S. Yang, Sharon Chinthrajah, Thomas Hagan, Tyson H. Holmes, Koji Abe

David Geffen School of Medicine at the University of California Los Angeles, Los Angeles CA 90095, USA: Elaine F. Reed, Joanna Schaenman, Ramin Salehi-Rad, Adreanne M. Rivera, Harry C. Pickering, Subha Sen, David Elashoff, Dawn C. Ward, Jenny Brook, Estefania Ramires-Sanchez, Megan Llamas, Claudia Perdomo, Clara E. Magyar, Jennifer Fulcher

University of California San Francisco, San Francisco, CA 94115, USA: David J. Erle, Carolyn S. Calfee, Carolyn M. Hendrickson, Kirsten N. Kangelaris, Viet Nguyen, Deanna Lee, Suzanna Chak, Rajani Ghale, Ana Gonzalez, Alejandra Jauregui, Carolyn Leroux, Luz Torres Altamirano, Ahmad Sadeed Rashid, Andrew Willmore, Prescott G. Woodruff, Matthew F. Krummel, Sidney Carrillo, Alyssa Ward, Charles R. Langelier, Ravi Patel, Michael Wilson, Ravi Dandekar, Bonny Alvarenga, Jayant Rajan, Walter Eckalbar, Andrew W. Schroeder, Gabriela K. Fragiadakis, Alexandra Tsitsiklis, Eran Mick, Yanedth Sanchez Guerrero, Christina Love, Lenka Maliskova, Michael Adkisson, Aleksandra Leligdowicz, Alexander Beagle, Arjun Rao, Austin Sigman, Bushra Samad, Cindy Curiel, Cole Shaw, Gayelan Tietje-Ulrich, Jeff Milush, Jonathan Singer, Joshua J. Vasquez, Kevin Tang, Legna Betancourt, Lekshmi Santhosh, Logan Pierce, Maria Tecero Paz, Michael Matthay, Neeta Thakur, Nicklaus Rodriguez, Nicole Sutter, Norman Jones, Pratik Sinha, Priya Prasad, Raphael Lota, Saurabh Asthana, Sharvari Bhide, Tasha Lea, Yumiko Abe-Jones

Yale School of Medicine, New Haven, CT 06510, USA: David A. Hafler, Ruth R. Montgomery, Albert C. Shaw, Steven H. Kleinstein, Jeremy P. Gygi, Dylan Duchen, Shrikant Pawar, Anna Konstorum, Ernie Chen, Chris Cotsapas, Xiaomei Wang, Charles Dela Cruz, Akiko Iwasaki, Subhasis Mohanty, Allison Nelson, Yujiao Zhao, Shelli Farhadian, Hiromitsu Asashima, Omkar Chaudhary, Andreas Coppi, John Fournier, M. Catherine Muenker, Khadir Raddassi, Michael Rainone, William Ruff, Syim Salahuddin, Wade L. Shulz, Pavithra Vijayakumar, Haowei Wang, Esio Wunder Jr., H. Patrick Young, Albert I. Ko, Gisela Gabernet

Yale School of Public Health, New Haven, CT 06510, USA: Denise Esserman, Leying Guan, Anderson Brito, Jessica Rothman, Nathan D. Grubaugh, Kexin Wang, Leqi Xu

Baylor College of Medicine and the Center for Translational Research on Inflammatory Diseases, Houston, TX 77030, USA: David B. Corry, Farrah Kheradmand, Li-Zhen Song, Ebony Nelson

Oklahoma University Health Sciences Center, Oklahoma City, OK 73104, USA: Jordan P. Metcalf, Nelson I. Agudelo Higuita, Lauren A. Sinko, J. Leland Booth, Douglas A. Drevets, Brent R. Brown

University of Arizona, Tucson AZ 85721, USA: Monica Kraft, Christian Bime, Jarrod Mosier, Heidi Erickson, Ron Schunk, Hiroki Kimura, Michelle Conway, Dave Francisco, Allyson Molzahn, Connie Cathleen Wilson, Ron Schunk, Trina Hughes, Bianca Sierra

University of Florida, Gainesville, FL 32611, USA: Mark A. Atkinson, Scott C. Brakenridge, Ricardo F. Ungaro, Brittany Roth Manning, Lyle Moldawer

University of Florida, Jacksonville, FL 32218, USA: Jordan Oberhaus, Faheem W. Guirgis

University of South Florida, Tampa FL 33620, USA: Brittney Borresen, Matthew L. Anderson

The University of Texas at Austin, Austin, TX 78712, USA: Lauren I. R. Ehrlich, Esther Melamed, Cole Maguire, Dennis Wylie, Justin F. Rousseau, Kerin C. Hurley, Janelle N. Geltman, Nadia Siles, Jacob E. Rogers, Pablo Guaman Tipan

We thank the participants of the study for their voluntary enrollment and contribution of samples for this work. See the supplement for details on the IMPACC Network. We acknowledge the assistance of the following individuals: Sanya Thomas, Mitchell Cooney, Shun Rao, Sofia Vignolo, and Elena Morrocchi (all from the CDCC); Arash Naeim, Marianne Bernardo, Sarahmay Sanchez, Shannon Intluxay, Clara Magyar, Jenny Brook, Estefania Ramires-Sanchez, Megan Llamas, Claudia Perdomo, Clara E. Magyar, and Jennifer A. Fulcher (all from the David Geffen School of Medicine at UCLA); members of the UCLA Center for Pathology Research Services and the Pathology Research Portal; M. Catherine Muenker, Dimitri Duvilaire, Maxine Kuang, William Ruff, Khadir Raddassi, Denise Shepherd, Haowei Wang, Omkar Chaudhary, Syim Salahuddin, John Fournier, Michael Rainone, and Maxine Kuang (all from the Yale School of Medicine). We thank the leadership of Boston Children's Hospital including Drs. Wendy Chung, Gary Fleisher and Kevin Churchwell for their support for the Precision Vaccines Program.

Supplemental Conflicts of Interest:

IMPACC Network Competing Interests

The Icahn School of Medicine at Mount Sinai has filed patent applications related to SARS-CoV-2 serological assays, NDV-based SARS-CoV-2 vaccines, influenza virus vaccines, and therapeutics, listing Florian Krammer and Viviana Simon as co-inventors. Mount Sinai has spun out Kantaro to market SARS-CoV-2 serological tests and Castlevax to develop SARS-CoV-2 vaccines, with Florian Krammer as co-founder and scientific advisory board member of Castlevax. Florian Krammer has consulted for Merck, Curevac, Seqirus, GSK, Pfizer, 3rd Rock Ventures, Sanofi, Gritstone, and Avimex. His laboratory collaborates with Dynavax on influenza vaccine development and with VIR on influenza virus therapeutics development. Ofer Levy is a named inventor on patents held by Boston Children's Hospital related to vaccine adjuvants and human in vitro platforms that model vaccine action. His laboratory has received research support from GlaxoSmithKline (GSK) and Pfizer, he is a consultant to GSK and Sanofi, and he is a co-founder and advisor to ARMR Sciences (formerly Ovax, Inc.), which develops technologies to detect illicit substances and prevent overdose. Charles Cairns consults for bioMérieux and receives grant funding from the Bill & Melinda Gates Foundation. James A. Overton is a consultant at Knocean Inc. Jessica Lasky-Su is a scientific advisor for Precion Inc. Scott R. Hutton, Greg Michelloti, and Kari Wong are employees of Metabolon Inc. Vicki Seyfer-Margolis is employed by MyOwnMed. Nadine Rouphael reports grants or contracts with Merck, Sanofi, Pfizer, Vaccine Company, and Immorna. She has served on data safety monitoring boards for Moderna, Sanofi, Seqirus, Pfizer, EMMES, ICON, BARDA, and CyanVan Micron. She has received travel support from Sanofi and Moderna and honoraria from Virology Education and Krog Consulting. Chris Cotsapas is employed by Vesalius Therapeutics. Adeeb Rahman is employed by Immunai Inc. Steven Kleinstein consults for Peraton related to the ImmPort data repository. Nathan Grubaugh consults for Tempus Labs and the National Basketball Association. Akiko Iwasaki consults for 4BIO, Blue

Willow Biologics, Revelar Biotherapeutics, RIGImmune, Xanadu Bio, and Paratus Sciences. Monika Kraft receives research funding from NIH, ALA, Sanofi, and AstraZeneca for asthma research. She consults for AstraZeneca, Sanofi, Chiesi, and GSK for severe asthma and is co-founder and CMO of RaeSedo, Inc., developing peptidomimetics for inflammatory lung disease. Esther Melamed receives research funding from Babson Diagnostics, honoraria from the Multiple Sclerosis Association of America, and has served on advisory boards for Genentech, Horizon, Teva, and Viela Bio. Carolyn Calfee receives research funding from NIH, FDA, DOD, Roche-Genentech, and Quantum Leap Healthcare Collaborative and consults for Janssen, Vasomune, Gen1e Life Sciences, NGMBio, and Cellenkos. Wade Schulz has collaborated with the Shenzhen Center for Health Information and the National Center for Cardiovascular Diseases in Beijing, is a technical consultant for Hugo Health, co-founder of Refactor Health, and has received grants from Merck and Regeneron Pharmaceuticals for COVID-19 research. Grace A. McComsey receives research grants from Redhill, Cognivue, Pfizer, and Genentech and consults for Gilead, Merck, and ViiV/GSK. Linda N. Geng receives research funding from Pfizer, Inc., through her institution. Catherine Hough receives research support from NIH and CDC. David Hafler has received research funding from Bristol-Myers Squibb, Novartis, Sanofi, and Genentech and consults for Bayer Pharmaceuticals, Repertoire Inc., Bristol Myers Squibb, Compass Therapeutics, EMD Serono, Genentech, Novartis Pharmaceuticals, and Sanofi Genzyme.

Supplemental Figure 1. Generation and validation of BWZ.CD16A reporter cells. A) BWZ cells expressing chimeric CD16A-z receptors were generated and confirmed to express cell surface receptor using flow cytometry. **B**) Stimulation of BWZ.CD16A reporter cells using platebound purified antibodies of shown isotypes. **C**) Titration curve showing dose response of antibody coated Spike RBD stimulating BWZ.CD16A reporter cells.

Supplemental Figure 2. The CD16A V176 allele remains associated with lower anti-SARS-CoV-2 titers and improved respiratory status at visit 4. A) Respiratory status breakdown by CD16A genotype at visit 4 (14 days from hospital admission), P=0.025. 1= not hospitalized + no limitations, 2= not hospitalized + limitations, 3= hospitalized + no O2, 4= hospitalized + required O2, 5= non-invasive ventilation or high flow O2, 6= mechanical ventilation or ECMO, 7= death. **B)** Mortality stratified by CD16A genotype (OR=0.963, 95% CI=0.726-1.268, P=0.788). **C-D)** Visit 4 antibody titers of RBD **(C)** and spike **(D)** stratified by CD16A genotype (P=0.01 and 0.006, respectively). **E)** Viral rpM at visit 4 split by CD16A genotype (P=0.246).

Supplemental Figure 3. Individuals homozygous for the CD16A V176 allele have a weakened correlation between anti-spike titers and viral rpM as well as viral rpM and COVID-19 severity. A-B) Correlation plots for viral rpM and spike titers **(A)** or RBD titers **(B)** split across CD16A genotypes. Black line is for that specific genotype, and red line is all patients together. Dashed lines are 95% confidence intervals. **C)** Box-and-whisker plots of viral rpM for mild (TG123) versus severe (TG45) COVID-19 trajectory groups split by CD16A genotype.

Supplementary Figure 4. No difference in blood NK cell counts based on CD16A genotype in the IMPACC cohort. A-E) CyTOF was performed on whole blood of N=788 participants and immune cell counts were determined. **(A)** CD56low CD16hi NK cell counts (P=0.095), **(B)** CD56hi CD16low NK cell counts (P=0.086), **(C)** monocyte counts (P=0.034), **(D)** neutrophil counts (P=0.618), and **(E)** CD39high CD4+ Treg counts (P=0.192) were recorded split by CD16A genotype.

Supplemental Figure 1

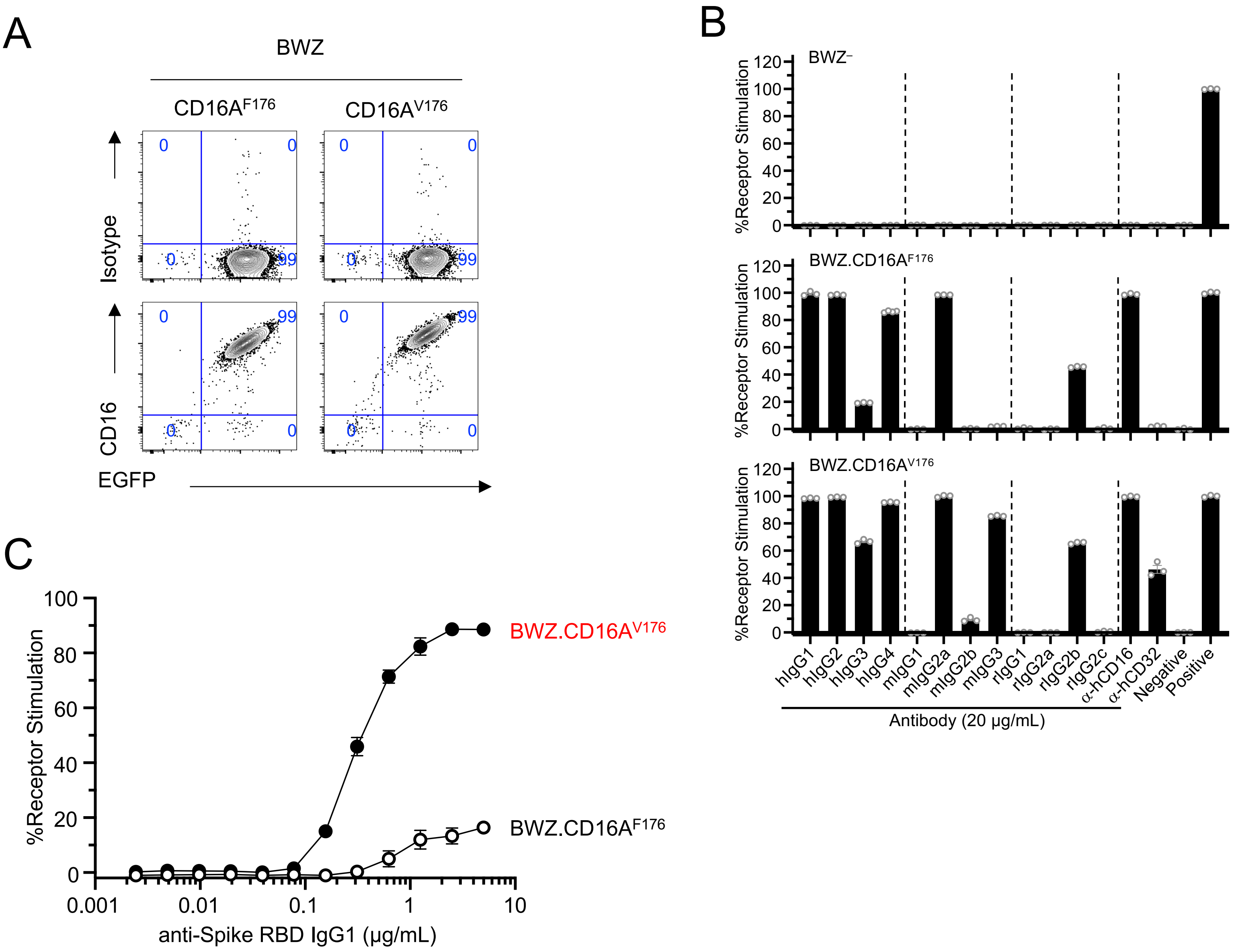

Supplemental Figure 1. Generation and validation of BWZ.CD16A reporter cells. A) BWZ cells expressing chimeric CD16A-ζ receptors were generated and confirmed to express cell surface receptor using flow cytometry. **B**) Stimulation of BWZ.CD16A reporter cells using platebound purified antibodies of shown isotypes. **C**) Titration curve showing dose response of antibody coated Spike RBD stimulating BWZ.CD16A reporter cells.

Supplemental Figure 2

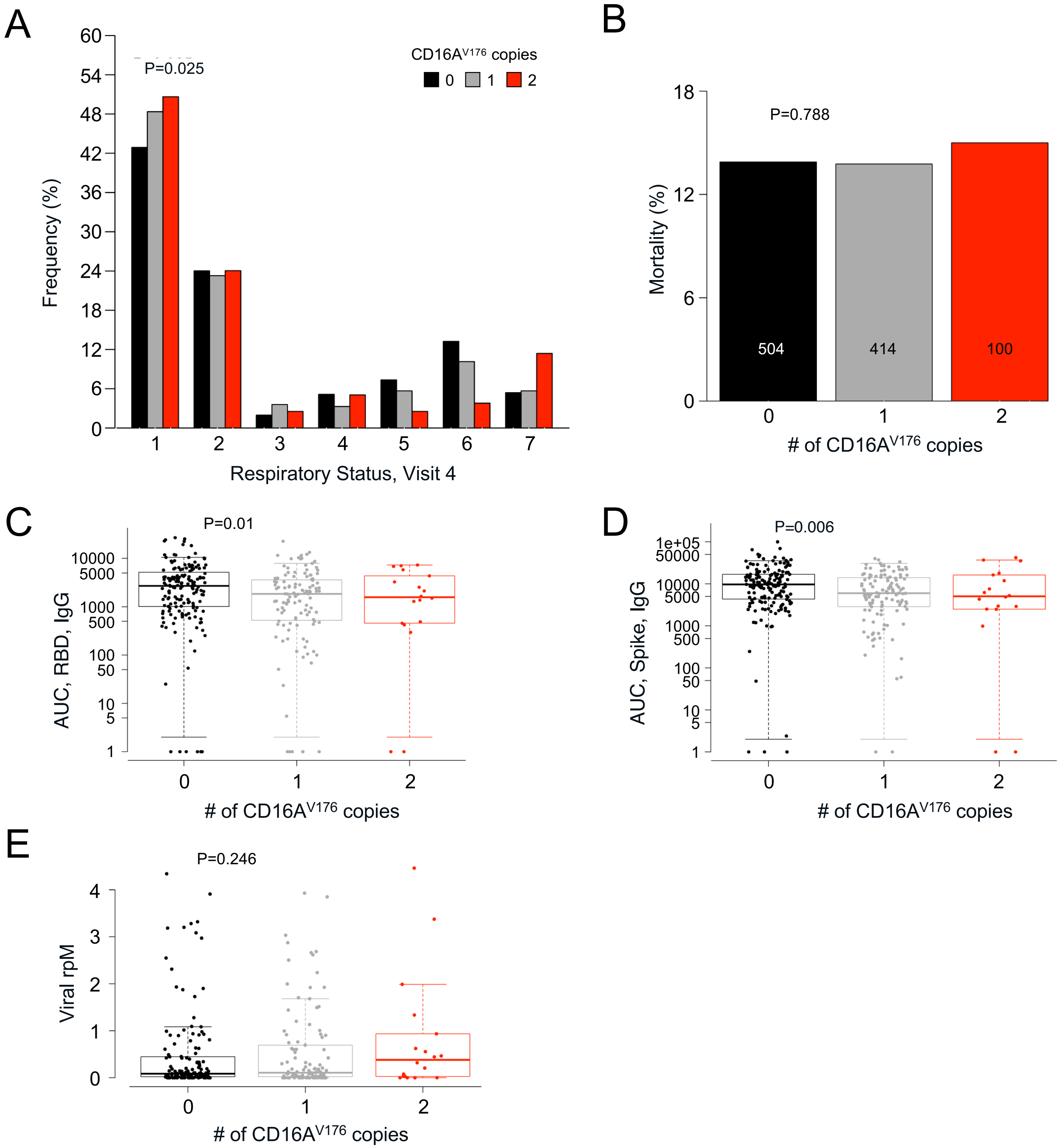

Supplemental Figure 2. The CD16A^{V176} allele remains associated with lower anti-SARS-CoV-2 titers and improved respiratory status at visit 4. A) Respiratory status breakdown by CD16A genotype at visit 4 (14 days from hospital admission), P=0.025. 1= not hospitalized + no limitations, 2= not hospitalized + limitations, 3= hospitalized + no O_2, 4= hospitalized + required O_2, 5= non-invasive ventilation or high flow O_2, 6= mechanical ventilation or ECMO, 7= death. **B)** Mortality stratified by CD16A genotype (OR=0.963, 95% CI=0.726-1.268, P=0.788). **C-D)** Visit 4 antibody titers of RBD **(C)** and spike **(D)** stratified by CD16A genotype (P=0.01 and 0.006, respectively). **E)** Viral rpM at visit 4 split by CD16A genotype (P=0.246).

Supplemental Figure 3

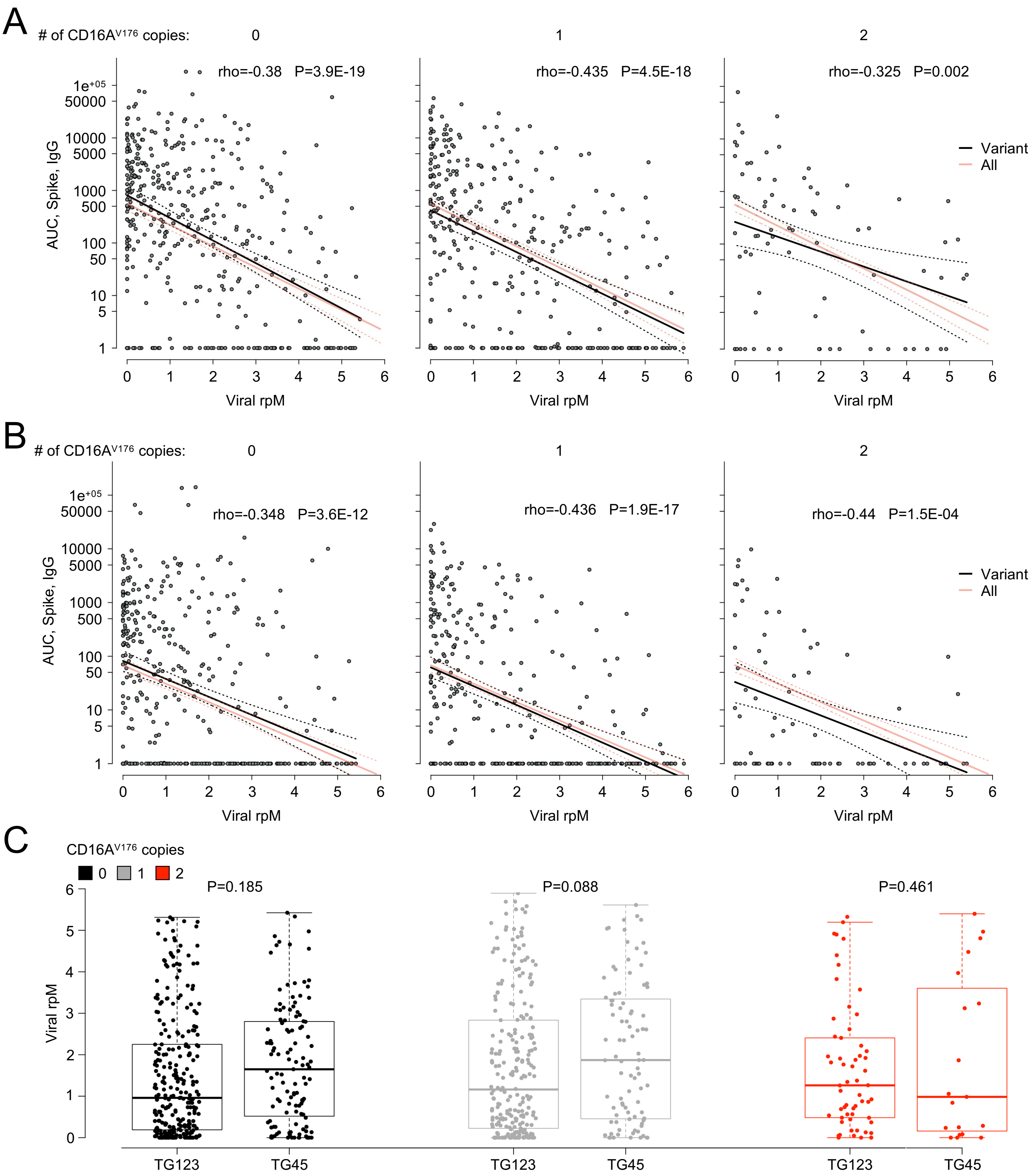

Supplemental Figure 3. Individuals homozygous for the CD16A^{V176} allele have a weakened correlation between anti-spike titers and viral rpM as well as viral rpM and COVID-19 severity. A-B) Correlation plots for viral rpM and spike titers **(A)** or RBD titers **(B)** split across CD16A genotypes. Black line is for that specific genotype, and red line is all patients together. Dashed lines are 95% confidence intervals. **C)** Box-and-whisker plots of viral rpM for mild (TG123) versus severe (TG45) COVID-19 trajectory groups split by CD16A genotype.

Supplemental Figure 4

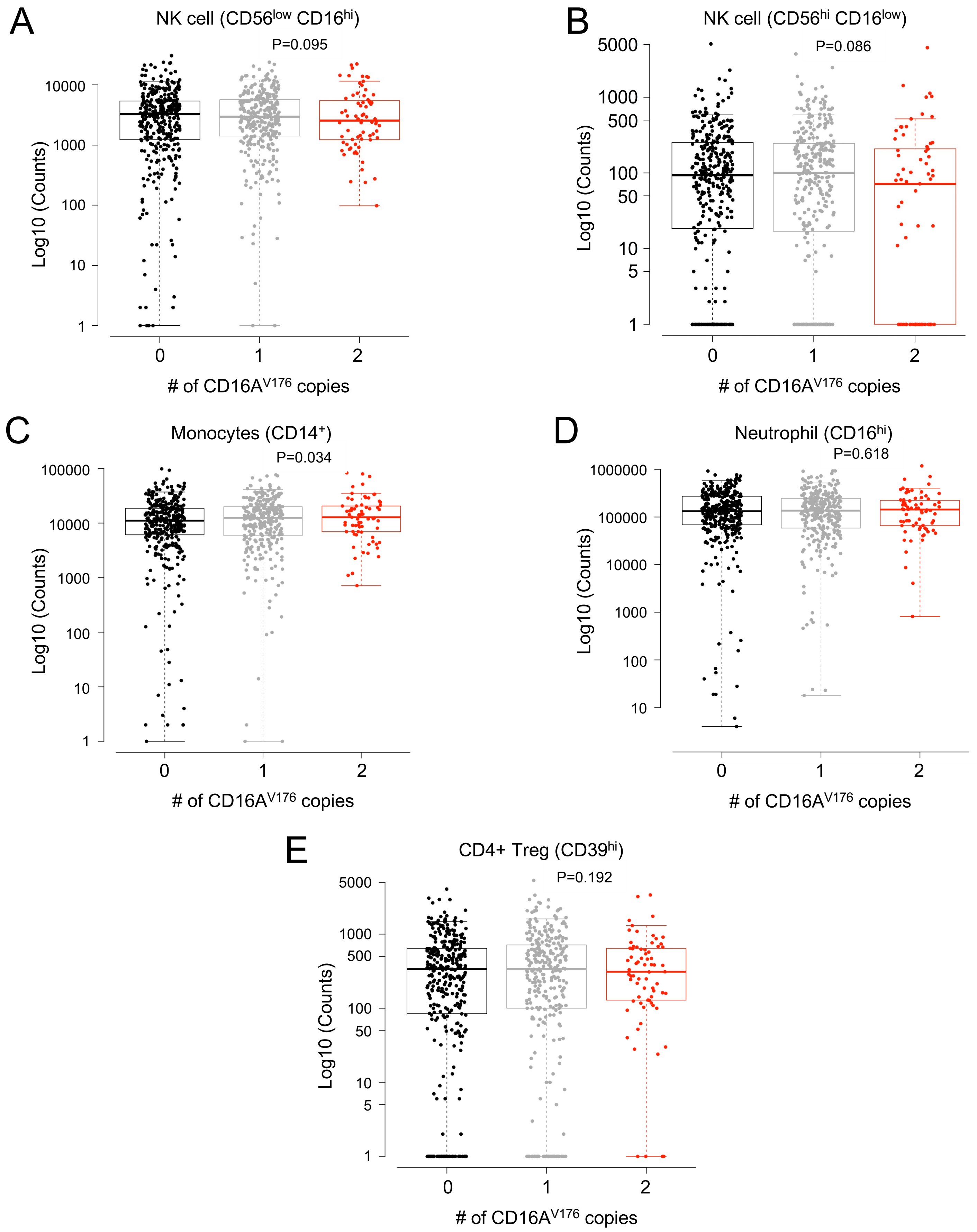

Supplementary Figure 4. No difference in blood NK cell counts based on CD16A genotype in the IMPACC cohort. A-E) CyTOF was performed on whole blood of N=788 participants and immune cell counts were determined. **(A)** CD56^{low} CD16^{hi} NK cell counts (P=0.095), **(B)** CD56^{hi} CD16^{low} NK cell counts (P=0.086), **(C)** monocyte counts (P=0.034), **(D)** neutrophil counts (P=0.618), and **(E)** CD39^{high} CD4+ Treg counts (P=0.192) were recorded split by CD16A genotype.
